# Zebrafish Collagen Type I: Molecular and Biochemical Characterization of the Major Structural Protein in Bone and Skin

**DOI:** 10.1038/srep21540

**Published:** 2016-02-15

**Authors:** C. Gistelinck, R. Gioia, A. Gagliardi, F. Tonelli, L. Marchese, L. Bianchi, C. Landi, L. Bini, A. Huysseune, P. E. Witten, A. Staes, K. Gevaert, N. De Rocker, B. Menten, F. Malfait, S. Leikin, S. Carra, R. Tenni, A. Rossi, A. De Paepe, P. Coucke, A. Willaert, A. Forlino

**Affiliations:** 1 Center for Medical Genetics Ghent, Ghent University, Ghent, Belgium; 2 Department of Molecular Medicine, Biochemistry Unit, University of Pavia, Pavia, Italy; 3 Functional Proteomics Lab., Department of Life Sciences, University of Siena, Siena, Italy; 4 Biology Department, Ghent University, Ghent, Belgium; 5 Department of Medical Protein Research, VIB, Ghent, Belgium; 6 Department of Biochemistry, Ghent University, Ghent, Belgium; 7 Eunice Kennedy Shriver National Institute of Child Health and Human Development, National Institutes of Health, Bethesda, MD, USA; 8 Department of Biosciences, University of Milano, Milan, Italy

## Abstract

Over the last years the zebrafish imposed itself as a powerful model to study skeletal diseases, but a limit to its use is the poor characterization of collagen type I, the most abundant protein in bone and skin. In tetrapods collagen type I is a trimer mainly composed of two α1 chains and one α2 chain, encoded by *COL1A1* and *COL1A2* genes, respectively. In contrast, in zebrafish three type I collagen genes exist*, col1a1a*, *col1a1b* and *col1a2* coding for α1(I), α3(I) and α2(I) chains. During embryonic and larval development the three collagen type I genes showed a similar spatio-temporal expression pattern, indicating their co-regulation and interdependence at these stages. In both embryonic and adult tissues, the presence of the three α(I) chains was demonstrated, although in embryos α1(I) was present in two distinct glycosylated states, suggesting a developmental-specific collagen composition. Even though in adult bone, skin and scales equal amounts of α1(I), α3(I) and α2(I) chains are present, the presented data suggest a tissue-specific stoichiometry and/or post-translational modification status for collagen type I. In conclusion, this data will be useful to properly interpret results and insights gained from zebrafish models of skeletal diseases.

In the last years, the small fresh water teleost *Danio rerio* (zebrafish) imposed itself as a good model for the study of heritable skeletal diseases^1^. Several zebrafish mutants that accurately model human skeletal diseases have been reported, such as *frilly fins*, *microwaved* and *chihuahua*, all reproducing the brittle bone disease osteogenesis imperfecta^2^,^3^. Furthermore, the molecular basis and the key regulators of skeletogenesis were demonstrated to be highly conserved between zebrafish and mammals^4^,^5^. The embryo transparency together with the availability of many zebrafish lines expressing fluorescent proteins under the control of skeletal specific promoters facilitated the investigation of early skeletal development^6^, whereas the generation of mutant lines for specific cartilage and bone proteins favoured the understanding of late skeletogenesis events^7^,^8^. Furthermore, the TALEN and CRISPR/Cas-based methods for genome editing developed in the last years allow the rapid generation of new zebrafish skeletal mutants^9^. Of relevance, since zebrafish are aquatic animals, the weight-bearing demand on the skeleton is reduced, therefore many bone mutant lines survive far longer than mouse models allowing characterization from embryonic to adult stages. Finally, due to their ability to produce large clutches of transparent embryos and their small size, high-throughput drug screening is also easier and economically advantageous using zebrafish instead of murine models and this is particularly appealing for the identification of osteogenic and osteotoxic molecules^10^.

The most abundant protein present in bone is collagen type I, but its features in zebrafish are still puzzling the research field. More insights into the stoichiometry and localization of collagen type I during zebrafish development and in adult tissues are necessary to properly interpret findings gained from zebrafish models for skeletal diseases.

Type I collagen is a member of the fibrillar collagen family, since it is characterized by a distinctive uninterrupted “collagenous” domain, a right handed triple helix formed by three left handed α chains supercoiled around a central axis^11^. In the endoplasmic reticulum, type I collagen is synthesized as a pro-collagen molecule characterized by the presence of N- and C-propeptides flanking the triple helical region via two short telopeptides (N- and C- telopeptide). In particular, the C-propeptide contains a variable sequence of 15 residues, known as the chain recognition region, necessary for chain identification, association and stoichiometry^12^. In the C-propeptide there is also a series of conserved cysteine residues involved in the formation of intra- and inter-chain disulphide bonds necessary for the correct conformation of the propeptide itself and for the covalent stabilization of the newly forming triple helix molecule^13^. Following secretion, specific peptidases are responsible for the removal of the N- and C-propeptides, allowing the mature triple helix to self-assemble into fibrils. Fibrils are stabilized by cross-links occurring between specific lysine and hydroxylysine residues located in the telopeptides (9^N^ and 16^C^ in α1 and 5^N^ in α2) and at both the extremities of the triple helical domain (at positions 87 and 930 in α1 and 87 and 933 in α2)^14^. These residues are involved in the formation of both inter- and intra-molecular covalent bonds. Structural or quantitative defects in type I collagen cause osteogenesis imperfecta (OI), also known as brittle bone disease, an inherited skeletal disorder that is mainly characterized by increased bone fragility, fracture risk and reduced bone strength^15^. Several studies have also demonstrated the association between variants identified in the genes encoding for collagen type I and bone density and risk of fractures in patients with senile osteoporosis^16^.

In all tetrapods type I collagen is mainly composed of two identical α1 chains and one α2 chain, encoded by *COL1A1* and *COL1A2* genes, respectively. However, homotrimeric type I collagen, consisting of three α1 chains, was described as a minor component of human skin^17^, present in chick embryos^18^ and associated with the extracellular matrix produced by cancer cells^19^. In many teleosts, including zebrafish, the presence of a distinct gene coding for a third α(I) chain, named α3, has been reported^20^. Based on amino acid sequence and on peptide analysis, the described third chain of type I collagen was shown to be phylogenetically more similar to the α1(I) than to the α2(I) chain. The similarity between α1(I) and α3(I) suggested that the genes for both proteins probably originated from the duplication of an ancestor α(I) coding gene during the whole genome duplication event that occurred ~320 mya at the basis of teleost evolution^21^. The first description of the presence at protein level of the α3(I) chain in fishes dates back to 1965^22^, when it was identified in the collagen type I extracted from the Atlantic cod (*Gadus morhua*) skin. Several years later Kimura and colleagues classified teleosts in three groups based on the α3 properties of acid soluble collagen type I^23^. A first teleost group, including ayu, flying fish and saury, missed this chain; a second group, including salmon and rainbow trout, was characterized by the presence of an α3 chain co-eluting with α2 by chromatographic separation and co-migrating with α1 by electrophoresis; finally, in a third group including most of the teleosts, α3 was co-migrating with α1 both by chromatography and by electrophoresis^24^. In all the analysed fishes the suggested stoichiometry for the three α(I) chains was 1:1:1. Interestingly, in acid soluble collagen type I obtained from carp and tilapia skin, α3 was present in very low amount, suggesting the presence of two different type I collagen heterotrimers, (α1)_2_α2 and α1α2α3^24^. The first complete primary structure of a teleost type I collagen was identified in 2001 in rainbow trout, and a 1:1:1 stoichiometry for the three chains was demonstrated in skin, whereas muscle type I collagen was mainly constituted by α1 and α2 with a stoichiometry of (α1)_2_α2^25^.

In zebrafish the existence of three genes, *col1a1a*, *col1a1b* and *col1a2* coding for collagen type I α1, α3 and α2 chains was reported^20^, but to date neither information about their expression during development nor biochemical data about the molecular composition are available. In this work, we report for the first time an extensive molecular and biochemical analysis of zebrafish collagen type I. A similar spatio-temporal expression pattern for c*ol1a1a, col1a1b* and *col1a2* was demonstrated starting from the oocyte stage until adult age. The existence of the α3 chain was demonstrated at the protein level both in zebrafish embryos and in adult skin, scales and bone. In embryos α1(I) was present in two distinct post-translationally glycosylated states, suggesting a developmental or tissue specific type I collagen composition in zebrafish. In adult tissues no significant differences were observed in type I collagen in terms of electrophoretic migration, amino acid composition and denaturation temperature and the prevalence of a 1:1:1 ratio for α1(I), α2(I) and α3(I) was suggested.

## Results

### Similarity and differences between zebrafish and mammal type I procollagen

**Figure 1:**
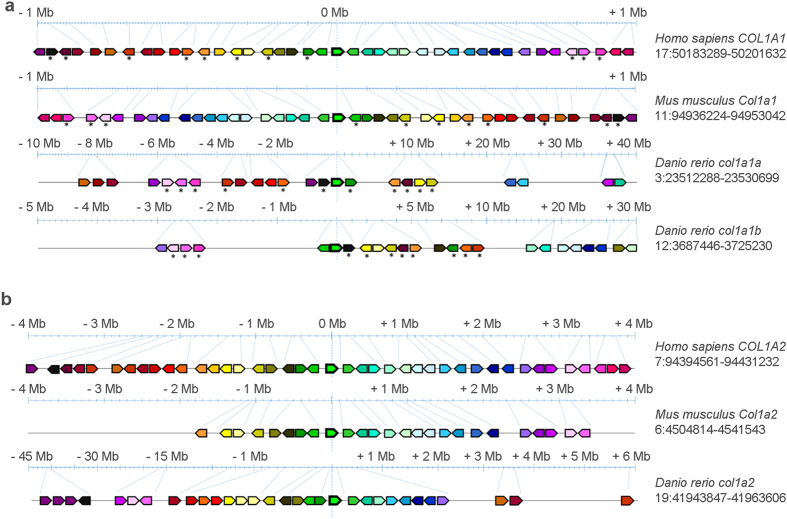
Synteny maps comparing the genes flanking type I collagen loci among human (*H. sapiens*), mouse (*M. musculus*) and zebrafish (*D. rerio*) chromosomes. The maps were obtained using the genome browser Genomicus. The human genes were used as roots. The position of the genes (Mb) relative to the investigated locus is based on Ensemble database and shown on top of the chromosome line. The exact chromosomal position of all the conserved genes is reported in the Supplementary Tables S1 and S2. The direction of the arrows indicates the gene orientation in respect to the reference gene. (**a**) Synteny map for *COL1A1* locus. Eleven genes flanking human and murine *COL1A1* are present in the zebrafish genome flanking both *col1a1a* and *col1a1b* loci (asterisk). (**b**) Synteny map revealed 27 conserved genes flanking human *COL1A2* that have orthologs in zebrafish chromosome 19 surrounding *col1a2*.

A synteny analysis of the genes surrounding the zebrafish collagen type I genes *col1a1a, col1a1b* and *col1a2* was first undertaken. The analysis of the chromosomal regions surrounding the zebrafish *col1a1a* and *col1a1b*, as well as the mouse and human *COL1A1* genes, revealed a shared synteny, supporting the presence of a common ancestral chromosomal origin (Fig. 1a). However, the number of the syntenic genes flanking human/mouse *COL1A1* and zebrafish *col1a1a* and *col1a1b* is limited and also their location and arrangement are different. (Fig. 1a, Supplementary Table S1).

A strong synteny was identified between the genomic regions surrounding the zebrafish *col1a2* and human/murine *COL1A2* locus (Fig. 1b, Supplementary Table S2).

To evaluate the conservation of the type I collagen alpha chains we considered the whole chain sequence (proα1(I), proα2(I) and proα3(I)) as well as their different procollagen domains, namely the N-propeptide, N-telopeptide, triple helix, C-telopeptide and C-propeptide. Amino acid (AA) sequence alignments revealed that zebrafish proα1(I) and proα3(I) chains share 78% of AA identity and the same theoretical molecular weight (137 kDa) and isoelectric point (5.4) (Table 1). Moreover, zebrafish proα1(I) shows 77% and 76% conserved AA with human and murine proα1(I) chains respectively, while proα3(I) has 75% identity with both of them. Since zebrafish type I procollagen domains have not yet been described in literature, their boundaries were inferred from the alignment with the human and murine proα chains. The percentage of identity between zebrafish and human/mouse is high for all the analysed domains of the three chains (Table 1).

**Figure 2:**
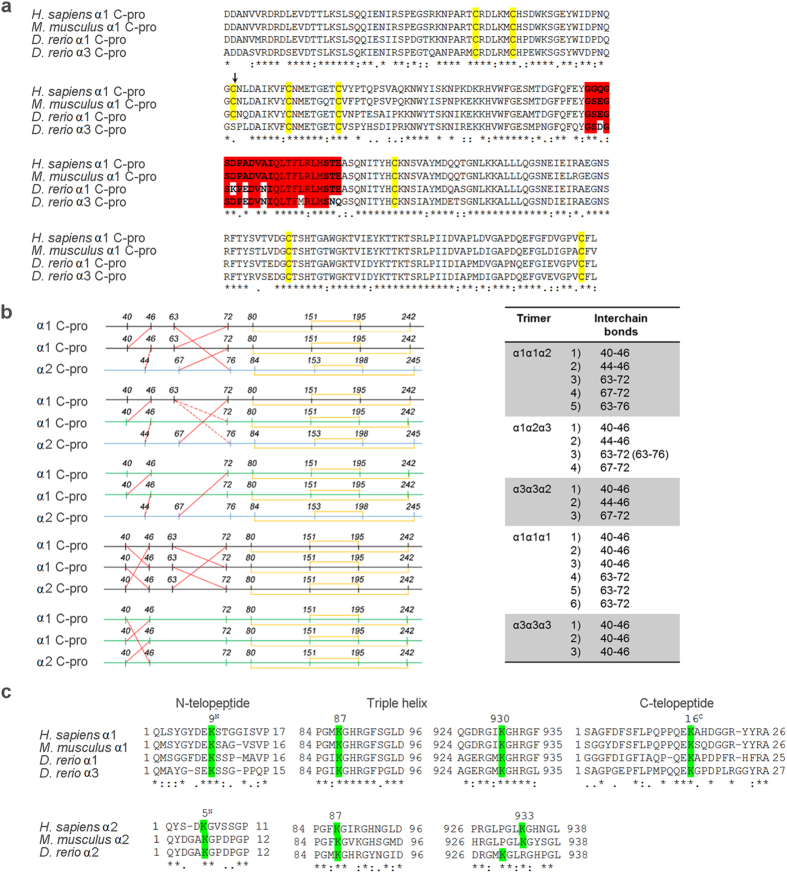
Comparison of conserved protein regions and amino acids with crucial function in type I procollagen chain assembly and extracellular collagen fibril formation. (**a**) Alignment of the α1 and α3 C-propeptide sequence in *D. rerio* with the α1 C-propeptide of human (*H. sapiens*) and mouse (*M. musculus*). In the recognition sequence (highlighted in red), responsible for the α chain assembly in a trimeric molecule, the α3 chain has 6 different AA when compared to human/mouse α1 whereas for α1 only 3 AA are different. The cysteine residues responsible for inter- and intra-chain disulfide bonds (yellow) are all conserved in zebrafish α1 but Cys63 (arrow), involved in inter-chain bonds, is missing in α3 and substituted with a Ser. (**b**) Representative pattern of the inter- and intra-chain disulfide bonds (in red and yellow respectively) in all the possible trimer compositions. (**c**) Lys/Hyl residues known to be involved in type I collagen cross-link stabilization are highlighted in green. The human/mouse Lys/Hyl α2(I) 933 is substituted in zebrafish α2 by an arginine, but a Lys/Hyl is present in position 930.

As expected, due to the obligatory presence of glycine every third amino acid, a high sequence identity could be found for the triple helical domain (80% for α1 and 75% for α3 with both human and murine α1 chains). A BLASTP analysis comparing the second and third amino acid of the collagenous triplets Gly-X-Y was performed. The identity was over 60% for the α1 and α3 chains and over 50% for the α2 chain (Table 1). These results suggest a relatively high level of conservation for the collagen-non collagenous protein binding domains, that are known to be essential for collagen type I synthesis, secretion and function. As expected, based on their relevance for collagen α chain recognition and folding, the highest identity is found in the C-propeptide domain (85% and 83% for proα1 compared to human and murine proα1 chains respectively, and 78% and 76% for proα3 in respect to human and murine proα1). Interestingly, in proα3 this latter domain, crucial for chain association in the trimer, lacks one of the 4 conserved cysteine residues involved in inter-chain bonds (Cys63 of the proα1(I) C-propeptide), whereas no difference was found in number and position of the cysteines involved in intra-chain disulfide bonds (Fig. 2a,b). Also, the chain recognition region is more divergent in proα3 than in proα1 when compared to mammalian proα1 (Fig. 2a, Table 1).

In the extracellular environment, type I collagen self-assembles into fibrils stabilized by inter- and intra-molecule covalent cross-links involving specific Lys/Hyl residues in the α1 and α2 chains^26^. All the Lys/Hyl residues involved in human/mouse collagen type I cross-links are conserved in zebrafish with the exception of the α2(I) Lys/Hyl 933 that is substituted by an arginine. However, a Lys/Hyl is present at position 930 in the zebrafish α2(I) collagen sequence (Fig. 2c).

An important feature in determining the stability of the triple helix is the number of GG + GGG and GPP amino acid repeats. Low number of GG + GGG and high number of GPP repeats generally increase the stability of the triple helix^27^. In zebrafish α3(I) the number of GG + GGG repeats is about two-fold higher than in zebrafish α1(I), and both chains contain more of these repeats with respect to human and murine α1(I) (Supplementary Table S3). Interestingly, the number of GPP repeats is slightly higher in zebrafish α1(I) than in zebrafish α3(I) and significantly lower in zebrafish α1(I) and α3(I) when compared to human and mouse α1(I). Similarly, in zebrafish α2(I) the number of GG + GGG repeats is higher than in human and mice α2(I), but there is no significant difference in the GPP contents.

### Spatiotemporal co-expression of zebrafish collagen type I genes in early development

**Figure 3:**
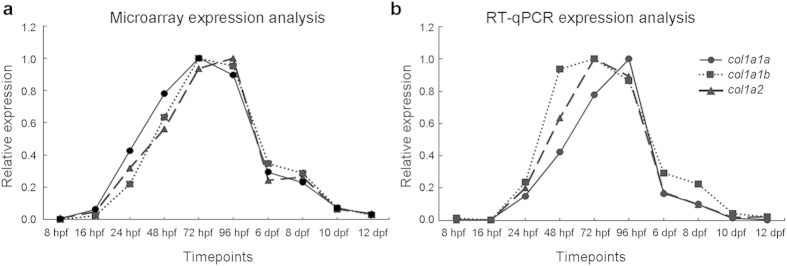
Expression of collagen type I genes in early zebrafish development. Relative expression values were normalized to maximum expression values for each gene and depicted in the y-axis. Both microarray (**a**) and RT-qPCR (**b**) analysis show similar expression patterns for *col1a1a*, *col1a1b* and *col1a2* with an expression peak for all three genes at 72–96 hpf.

In order to evaluate how the three collagen type I genes are transcribed during early zebrafish development, we assessed their relative expression at different time points during embryonic and larval development (8 hpf up to 12 dpf) by means of microarray and RT-qPCR analysis. The expression profiles of all three type I collagen genes were highly similar, with a peak in relative expression between 3 and 4 dpf (Fig. 3a,b), corresponding with the onset of bone formation in zebrafish embryos^28^. The expression of all three type I collagen genes was shown to be present, although at very low level, during the first hours post fertilization (Supplementary Fig. 1).

**Figure 4:**
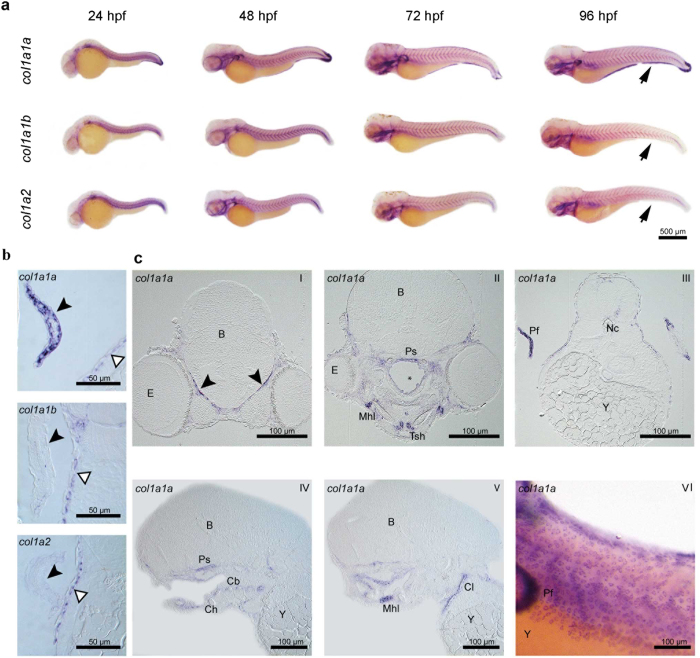
Whole mount *in situ* hybridization (WISH) for type I collagen genes in zebrafish embryos. (**a**) WISH for *col1a1a, col1a1b* and *col1a2* at different time points (24, 48, 72 and 96 hpf) of zebrafish development. Expression patterns are similar for all three type I collagen genes. However, expression of *col1a1a,* but not of *col1a1b* nor *col1a2,* was clearly noted in the fin fold (indicated by the arrowhead) and in the pectoral fins at 96 hpf. (**b**) Expression of *col1a1a*, *col1a1b* and *col1a2* at 96 hpf in the pectoral fin (black arrowheads). Only for *col1a1a* a clear signal could be detected. Note that basal epidermal cells are similarly labeled for all three collagen type I genes (open arrowheads). (**c**) Cross (CI-CIII) and sagittal (CIV-CV) sections of 96 hpf WISH specimens, showing details of expression of *col1a1a* in various cell or tissue types: meninges (CI, arrowheads), osteoblasts surrounding the parasphenoid bone (Ps, CII, CIV) and the cleithrum (Cl, CV), fibroblasts of the mandibulo-hyoid ligament (Mhl, CII, CV) and of the tendon of the sternohyoideus muscle (Tsh, CII) and distal epidermal/subepidermal layers of the pectoral fins (CIII). Mesenchymal cells around ceratobranchials 1–4 display a weak, and around ceratobranchial 5 a somewhat stronger signal (Cb 1–5, CIV). CVI shows the expression of *col1a1a* in the basal cells of the epidermis. Further abbreviations: B, brain; Ch, ceratohyal; E, eye; Nc, notochord; Pf, pectoral fin; Y, yolk. Cavity marked by * on CII is an artefact.

To further explore the spatio-temporal expression of the *col1a1a, col1a1b* and *col1a2* genes during embryonic development, whole mount *in situ* hybridization (WISH) was carried out at 24, 48, 72 and 96 hpf (Fig. 4a). At every time point, expression of all three genes could be detected in the epidermis. Interestingly, only *col1a1a* was shown to be expressed in the median fin fold and in the apical ectodermal ridge of the pectoral fin at 48, 72 and 96 hpf (Fig. 4a,b). Because several elements of the craniofacial skeleton appear at 96 hpf, hybridized embryos at this time point were processed for semi-thin sectioning to evaluate and compare tissue-specific expression in internal structures (Fig. 4c and Table 2). All three type I collagen genes are expressed in mesenchymal cells that have entered final differentiation. Thus, all three collagen I genes are expressed in the osteoblasts surrounding the first bones that ossify in the larva: i.e. parasphenoid, opercular bone and cleithrum^28^,^29^. Fibroblasts of tendons and ligaments are particularly strongly labelled, as are the myosepta, connective tissue that separates the consecutive myomeres (Fig. 4c). These signals are consistent with the involvement of these cells in the production of a collagen-rich extracellular matrix. Likewise, the somewhat stronger signal in the mesenchyme surrounding the ceratobranchial 5 compared to the other ceratobranchials, can be explained by the more precocious perichondral ossification of these elements as tooth-bearing pharyngeal jaws^30^.

### Collagen type I in zebrafish embryos

**Figure 5:**
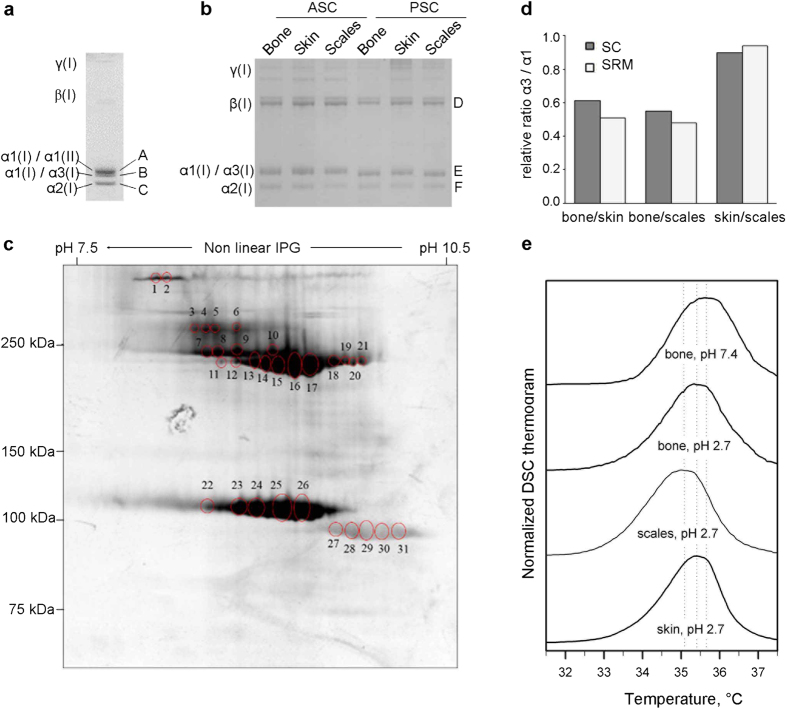
Analysis of type I collagen extracted from 48 hpf embryos and adult bone, skin and scales. (**a**) 1D SDS PAGE of pepsin extracted (PSC) type I collagen from 48 hpf embryos. The identification of the bands A, B and C was performed by mass spectrometry as detailed in Supplementary Table S4. The densitometric ratio between α1(I)-α1(II)/α2(I) was 1.70 ± 0.03, whereas the ratio α1(I)-α3(I)/α2(I) was 1.15 ± 0.09. (**b**) 1D SDS PAGE of acid soluble (ASC) and PSC from adult bone, skin and scales. The identity of the bands D, E and F was determined by mass spectrometry as detailed in Supplementary Table S6. (**c**) Representative 2D SDS PAGE of PSC from scales. Similar results were obtained for skin and bone. The identification of the spots 1 to 31 was performed by mass spectrometry as detailed in Supplementary Table S7. γ: collagen type I gamma trimers; β: collagen type I beta dimers. (**d**) Relative ratio α3(I)/α1(I) between bone, skin and scales as measured by selected reaction monitoring (SRM) and spectral counting (sc). In SRM, for each tissue three biological and two technical repeat measurements were analyzed. Spectral counts were measured in the same samples for one biological replicate. Skin and scales show high similarity (SRM multi Wilcoxon test p-value > 0.05) whereas bone is clearly distinguished (SRM multi Wilcoxon test p-value < 0.05) from these other tissues in their α3(I)/α1(I) ratio. The coefficient of variance calculated across all replicates, both technical and biological, per tissue type shows a good reproducibility in the measurement of α3(I)/α1(I) ratio (cv = 24%); (**e**) DSC thermograms of type I collagen purified from bone, skin and scales of adult zebrafish. DSC was performed in 0.2 M sodium phosphate, 0.5 M glycerol (pH 7.4) at 0.25 °C/minute heating rate (representative bone thermogram is shown, similar results were obtained from the other two tissues) and in 2 mM HCl, pH 2.7 at 0.25 °C/minute heating rate (the thermogram for the three tissues is represented).

Collagen type I was extracted by means of pepsin digestion and salt precipitation from 48 hpf embryos in order to evaluate its chain composition. Electrophoretic separation of collagen type I showed three monomeric α bands (Fig. 5a). Mass spectrometry analysis of these bands revealed that the upper one represented α1(I) chain co-migrating with α1(II), the middle band contained a mixture of α1(I) and α3(I) peptides and the lower band represented the α2(I) chain (Supplementary Table S4). The identification of α3(I) peptides demonstrated for the first time the translation of the *col1a1b* gene in zebrafish. The migration of the α1(I) chain in two distinct bands suggested the existence of two different levels of glycosylation for this chain, which could be attributed either to the presence of trimers with different stoichiometry and/or to a tissue specific glycosylation due to the tissue heterogeneity of the sample.

### Collagen type I stoichiometry in adult zebrafish tissues

SDS-PAGE of both completely (pepsin soluble, PSC) and uncompletely (acid soluble, ACS) cross-linked type I collagen extracted from internal (bone) and external (skin and scales) zebrafish tissues showed the canonical pattern reported for tetrapods, characterized by the presence of γ trimers, β dimers and two monomeric α bands^31^. As expected, acid soluble collagen migrated slightly slower than pepsin extracted collagen, due to the presence of N- and C-telopeptides (Fig. 5b). No obvious difference in size or relative quantity was detected among the bands of the three analysed tissues in both collagen fractions (Supplementary Table S5). Mass spectrometry of the α bands revealed in the upper band the co-migration of α1(I) and α3(I) and in the lower band the presence of α2(I) (Supplementary Table S6). The co-migration of α1(I) and α3(I) indicated their highly similar experimental molecular weight and glycosylation level. The densitometric ratio between the upper and lower α bands was around 2 for all three tissues, both in reducing and in non-reducing conditions (Supplementary Table S5), indicating there is generally twice as much α1(I) + α3(I) than α2(I). In the attempt to separate α1(I) from α3(I) chains based on their isoelectric point, 2D PAGE was performed. All the spots corresponding to γ trimers, β dimers and α monomers, resolved from each tissue, were analyzed by mass spectrometry (Fig. 5c). The identification of α1(I) and α3(I) chains from the same spot suggests similar pI values that prevent a proper resolution of these chains (Supplementary Table S7). Next, selected reaction monitoring (SRM) mass spectrometry was used to determine the abundance of α1(I) and α3(I) chains in gel-excised alpha bands from bone, skin and scales collagen extracts. A known concentration of synthetic peptide unique for α1(I) and α3(I) respectively was spiked-in, followed by comparison of the relative intensities of the endogenous and the synthetic spiked-in peptide. A pairwise comparison using the Wilcoxon rank sum test shows that the ratio α3(I)/α1(I) is equal between skin and scales (p-value = 0.059 > 0.05). The ratio in bone however is statistically different when compared to skin or scales (p-value = 2.2e^–10^ < 0.05), suggesting different amounts or molecular modifications of α1(I) and α3(I) in external (skin and scales) versus internal (bone) zebrafish tissues (Fig. 5d). Spectral counting of the unique peptides for α1(I) and α3(I), as measured in an LC-MS/MS run, revealed relative ratios of α3(I)/α1(I) among the three different tissues that were similar to the ones obtained by SRM mass spectrometry.

To estimate collagen type I chain stoichiometry in collagen extracts from bone, skin and scales, amino acid analysis was conducted. Based on the detected amount of Tyr residues (n = 2), that are theoretically absent in both α1(I) and α3(I) chains, a significant presence of α1(I) or α3(I) homotrimers could be excluded in all three tissues (Supplementary Table S8). The presence of a single α2 chain in the collagen type I trimers from the various tissues was assumed based on the fact that in tetrapods a single α2 chain is generally present in type I collagen molecules and based on the detected densitometric ratios between upper and lower α(I) bands in SDS-PAGE (Supplementary Table S5). This leaves us with three possible ratios for the three α chains (α1:α2:α3 in a 2:1:0 or 1:1:1 or 0:1:2 ratio). To discriminate among these possibilities, we considered the amino acids isoleucine and leucine since, based on theoretical amino acid composition, their ratio is over two-fold different between α1(I) (Ile/Leu = 0.92) and α3(I) (Ile/Leu = 0.36) chains. Based on the experimental ratio determined for these amino acids in the type I collagen from the three tissues (skin: 0.57 ± 0.05, bone: 0.54 ± 0.07 and scales: 0.55 ± 0.10), a 1:1:1 ratio for the three α chains in all tissues is inferred (Table 3). However, assuming that different trimers can be synthesized in various amounts, the existence of α1α2α3 alone or the presence of equal amounts of α1_2_α2 and α3_2_α2 or a combination of the three trimers cannot be excluded.

### Collagen type I purified from skin, bone and scales has similar thermal stability

A single denaturation peak with an apparent melting temperature T_m_ ~ 35 °C (±0.5 °C) was observed for zebrafish collagen type I in differential scanning calorimetry thermograms in 2 mM HCl, pH 2.7 at 0.25 °C/min heating rate (Fig. 5e). We observed no significant differences between acid soluble and pepsin treated collagens (data not shown) or between skin and bone collagens (Fig. 5e). The apparent T_m_ of scales collagen was ~0.4 °C lower, but within possible ~0.5 °C sample-to-sample variation (despite better than 0.1 °C reproducibility of the DSC instrument, sample-to-sample variations in DSC buffer and post-translational modification of collagen might affect T_m_ by up to ~0.5 °C). The apparent T_m_ at pH 7.4 (corrected by −1.7 °C to account for the stabilizing effect of phosphate-glycerol buffer compared to physiological saline^32^) was close to that at pH 2.7, also consistent with our previous observations for type I collagen from other species. The absence of any distinct peak at higher T_m_ in our samples further supports the absence of a detectable amount of homotrimers in zebrafish type I collagen. Higher T_m_ of α1(I) homotrimers compared to (α1)_2_α2 heterotrimers is expected to produce a pronounced second peak, e.g. observed in homotrimer/heterotrimer mixtures of mouse type I collagen^33^.

## Discussion

In this study, we performed a deep molecular and biochemical characterization of type I collagen in zebrafish. As collagen type I is the major structural protein in bone and skin, these data are of fundamental importance, not only to gain insights in the composition of the extracellular matrix of these tissues, but also to properly interpret results obtained from zebrafish models of heritable skin and bone diseases. Collagen type I mutations are responsible for various human diseases such as osteogenesis imperfecta^34^, Caffey disease (CAFFD)^35^, Ehlers-Danlos syndrome 1 (EDS1) and 7A (EDS7A)^36^. For some of these diseases zebrafish models already exist and TALEN and CRISPR/Cas genome editing now allows easy and efficient generation of novel mutants that could be used both to clarify pathophysiology and to test novel pharmacological approaches.

Type I collagen in tetrapods is mainly present as a heterotrimer constituted by two α1 and one α2 chains encoded by *COL1A1* and *COL1A2* genes respectively. Also in zebrafish, type I collagen is a trimer, but its α chain composition is puzzling since three types of α chains were identified, namely α1, α2 and α3, encoded by *col1a1a, col1a2* and *col1a1b* genes, respectively^20^. Our data demonstrated a co-regulation and interdependence of the three collagen type I genes during embryonic and larval zebrafish development. Expression analysis revealed peaking relative expression at 3–4 dpf of all three genes, coinciding with the formation of the first bony structures in the head of the zebrafish embryo, and thus emphasizing their relevance for bone formation during early development. Whole mount *in situ* hybridization (WISH) in zebrafish embryos and subsequent semi-thin sectioning showed overlapping expression patterns for all three genes, suggesting a strong co-expression and possibly co-regulation of type I collagen genes.

Expression of zebrafish collagen type I genes was mainly observed in the ectoderm, along developing bony elements, and in ligaments and tendons. This shows that the collagen type I genes are expressed in the same tissues as in humans, including bone and skin. Notably, several epithelial tissues expressed type I collagen genes. Expression of all three collagen genes produced a typical ‘leopard’ pattern in the skin. Sections revealed that transcripts were located in basal cells of the epidermis. Le Guellec *et al.* previously reported *col1a2* expression in the basal epidermis of zebrafish but did not consider the two other collagen genes^37^. Likely, all three type I collagen genes are involved in the production of the primary dermal stroma. Expression of *col1a1a*, but not of *col1a1b* nor *col1a2*, is also seen in the fin fold and in the distal margin of the pectoral fin. This signal is likely related to the formation of actinotrichia, non-calcified flexible rods that constitute the first fin skeleton formed during development. Durán *et al.* reported the expression of *col1a1a*, along with another type of collagen, *col2a1b*, and their contribution to actinotrichia formation^38^. Given the absence of *col1a1b* and *col1a2* expression, our findings also support the presence of α1(I) homotrimers in actinotrichia. Given that the signal at 96 hpf is clearly observed on both sides of the basement membrane, we propose that both epidermal and subepidermal cells synthesize *col1a1a* for integration in the actinotrichia, in support of the findings of Durán *et al.*^38^.

The expression, although at very low level, of all three collagen genes in zebrafish oocytes, indicates a role of maternal collagen type I transcripts in the earliest stages of development. In general, this study underlines the essential role for collagen type I during zebrafish embryogenesis, similarly as was already shown in other vertebrates; for instance lack of *Col1a1* expression in the homozygous *Col1a1*^*M*^*°*^*v13*^ mice is not compatible with life^39^.

In order to confirm that the annotated collagen type I genes in zebrafish are indeed the orthologues of the human/mouse collagen type I genes, we performed synteny analysis. Alignment of the genomic regions surrounding the zebrafish *col1a1a* and *col1a1b* genes with the regions flanking the human/murine *COL1A1* locus revealed a shared synteny, confirming the idea that the annotated collagen type I genes in zebrafish are indeed the orthologues of the human/mouse collagen type I genes and supporting the hypothesis that they all originate from a common ancestral gene, present in the last common ancestor of zebrafish and mammals. The same is valid for the zebrafish *col1a2* and the human *COL1A2* gene. On the other hand, the finding that only for a limited number of genes, flanking human and murine *COL1A1*, orthologues are present in the zebrafish genome flanking both *col1a1a* and *col1a1b* loci, shows a structural divergence of the chromosomal segments surrounding human and zebrafish type I collagen genes. Moreover, the different location and arrangement of the syntenic genes flanking *col1a1a* and *col1a1b* respectively suggested a divergent evolution of the corresponding chromosomal regions. Whether these differential chromosomal contexts also reflect specific divergent functions or expression patterns of the human versus zebrafish genes or the zebrafish *col1a1a* versus *col1a1b* genes can be suggested, but should be further investigated.

The high conservation of the sequence necessary for α chain recognition as well as of the C propeptide cysteine residues necessary for covalent inter-chain stabilization indicates that likely also in zebrafish, as in tetrapodes, the assembly of type I collagen trimer starts with C-propeptide association through a series of non-covalent interactions, that are then stabilized by the formation of inter-molecular disulphide bonds^40^,^41^. The lack of zebrafish α3 chain Cys63, that is known to participate in inter chain bounds, may have implication for chain stoichiometry. Further, the lysine residues known to be relevant for cross linking formation in type I collagen were identified in zebrafish, their conserved presence further support their relevance through evolution.

Interestingly, SDS PAGE of collagen extracted from zebrafish embryos followed by mass spectrometry analysis showed the presence of two α1(I) populations with a distinct molecular weight, one co-migrating with α1(II) and the other with α3(I). Since we obtained the embryonic collagen from whole body lysates it is likely that the variability in molecular weight of α1(I) is due to a difference in glycosylation level between different embryonic tissues, suggesting a developmental specific collagen composition. Possibly a fraction of collagen type I could be indeed constituted by α1(I) homotrimers coming from actinotrichia and maybe also lepidotrichia forming cells, characterized by overglycosylation of the α1(I) chains that migrate slower in SDS-PAGE. Indeed, only *col1a1a* and not *col1a1b* nor *col1a2* expression was detected in the fin folds by *in situ* hybridization. Due to the co-migration of α1(I) either with α1(II) or α3(I) chains it was impossible to determine exact ratios between these chains and hence make assumptions about the stoichiometry of chain association of embryonic collagen type I.

To gain insights into the composition of collagen type I in specific adult zebrafish tissues we analyzed collagen from skin, scales and bone by means of SDS-PAGE. We demonstrated an identical electrophoretic pattern for collagen type I obtained from all these tissues in 1D gels electrophoresis: a band containing co-migrating α1(I) and α3(I) and another band with lower molecular weight representing α2(I), with a stoichiometry of 2:1 between the two bands. 2D gel electrophoresis did not allow further separation of α1(I) and α3(I) spots. SRM and spectral counting mass spectrometry data shows a statistically significant higher α3(I)/α1(I) ratio in external (skin and scales) versus internal (bone) tissues pointing out to a tissue-specific collagen composition. Interestingly, similar findings have been previously reported in the Salmoniformes, a different order of Actinopterygii; in the skin of rainbow trout all three α(I) chains were detected, whereas in muscle, an internal tissue, only α1(I) and α2(I) were found to be present^25^. In contrast to the mass spectrometry data, our amino acid analysis of collagen type I extracted from zebrafish bone, skin and scales revealed an equal ratio for α1(I), α2(I) and α3(I) in all examined tissues. This apparent discrepancy between mass spectrometry and AA analysis could be explained considering the different nature of the analyzed samples in the different assays. Acid hydrolysis for amino acid analysis was performed on whole pepsin extracted collagen fraction that includes monomeric α chains as well as β dimers and γ trimers, whereas mass spectrometry data were obtained from electrophoretically separated monomeric α bands. It is known that collagen cross-links in soft tissue and bone are different as the lysyl hydroxylation level differs and a higher amount of immature cross-links is present in mineralized tissue^42^. Furthermore, at the supramolecular level, differences in organization and diameter of type I collagen fibrils have been reported between zebrafish bone, skin and scales^37^,^43^,^44^.Thus our data can indeed reflect such differences and pave the way for further investigation. Alternatively, we cannot exclude that differences in trypsin cleavage or site specific hydroxylation may influence the mass spectrometry data obtained, since it is known that collagen is not an easy target for proteolysis and that hydroxylation/glycosylation of bone and soft tissue collagen differ in tetrapods.

For the first time we were able to determine the temperature stability of zebrafish collagen type I that was characterized by apparent T_m_ ~35 °C in all three analyzed tissues. Human collagen type I is characterized by a T_m_ of 41.4 °C in skin and 43.3 °C in bone, due to the higher level of posttranslational modification in the latter^42^. In warm-blooded animals, increased post-translational modification and T_m_ of collagen from inner organs and tissues is likely related to higher physiological temperature of the latter^45^. Consistently, no such difference was present in cold-blooded zebrafish, which has no significant temperature gradient across the body. The lower T_m_ of zebrafish collagen type I compared to warm-blooded animals is consistent with generally observed correlation of collagen thermal stability with normal body temperature in different species^46^. It could be attributed to a higher number of GG + GGG motifs and a lower number of GPP triplets in zebrafish α(I) chains.

In conclusion, we provided a deep molecular and biochemical characterization of zebrafish collagen type I, the most abundant extracellular matrix protein in bone and skin. We demonstrated that, both in zebrafish embryos and adult tissues, three instead of two types of alpha chains contribute to the formation of the collagen type I triple helix, although the exact stoichiometry and post-translational modifications need to be further defined. These data will further pave the way to the use of zebrafish as a model for skin and skeletal diseases linked to abnormal type I collagen synthesis.

## Material and Methods

### Animals

Wild-type AB zebrafish were housed in ZebTEC semi-closed recirculation housing systems (Techniplast, Italy) and kept at a constant temperature (27–28 °C), pH (7.5) and conductivity (500 μS) on a 14/10 light/dark cycle. Embryos were collected by natural spawning and staged according to Kimmel^47^.

### Ethic Statement

All experiments were performed in accordance with the approved guidelines, in agreement with EU Directive 2010/63/EU for animals. The experimental protocols were approved by the Italian Ministry of Health (Approval Animal Protocol N. 1/2013) and by Ghent University Committee (Permit Number: ECD 15/22).

### *In silico* analysis

Synteny maps of the chromosomic regions surrounding type I collagen genes were constructed using human genes as reference by combining PhyloView and AlignView from Genomicus 81.01 (http://www.genomicus.biologie.ens.fr/genomicus-81.01/cgi-bin/search.pl)^48^ with Ensembl Comparative Genomics data. Zebrafish type I collagen domains were determined by alignment with *H. sapiens* and *M. musculus* sequences using ClustalW2^49^ and based on the annotation available from the osteogenesis imperfecta and Ehlers-Danlos syndrome variant database (www.le.ac.uk/genetics/collagen/)^50^. Percentage of identity was estimated using protein BLAST at NCBI.

### Collagen Extraction

Collagen was purified by a modification of the methods described in^51^. Briefly, bone, scales and skin obtained from adult fishes were delipidated in 0.1 N NaOH at 4 °C for 6 h, bone and scales were decalcified in 0.5 M EDTA, pH 7.4 at 4 °C for 48 h. Whole 2 days post-fertilization (dpf) embryos were manually dechorionated and homogenized in a Potter chamber in Phosphate Buffered Saline (PBS, Sigma Aldrich), pH 7.5 containing protease inhibitors (1.52 mg/mL EDTA, 1.57 mg/mL benzamidine, 0.25 mg/mL N-ethylmaleimide and 1 mM phenylmethanesulfonylfluoride). The acid-soluble collagen (ASC) was extracted by tissue incubation with 0.5 M acetic acid, while the pepsin-soluble fraction (PSC) was obtained digesting the remaining tissue with 0.1 mg/mL pepsin in 0.5 M acetic acid. Quantification was performed by measuring the 4-hydroxyproline amount^52^.

### Collagen Analysis by 1D-SDS-PAGE

Collagen (0.5 μg) was separated by 6% SDS-PAGE in the presence of 0.5 M urea. Gels were fixed in 45% methanol and 10% acetic acid, stained with 0.08 M picric acid, 0.04% Coomassie Brilliant Blue R250 (Sigma Aldrich) and destained in water. Versadoc3000 (BIO-RAD) and QuantityOne software (BIO-RAD) were used for gels digitalization and band densitometry.

### Collagen analysis by 2D-PAGE

2D-PAGE was performed using the Immobiline-polyacrylamide system as described in^53^. Isoelectric focusing (IEF) was carried out on preformed non-linear wide-range immobilized pH gradients (pH 6–11; 18 cm long IPG strips; GE Healthcare) and performed using the Ettan™ IPGphor™ III system (GE Healthcare). IPG strips were rehydrated with 350 μl of 8 M urea, 4% CHAPS, 1% dithioerythritol (DTE) and 2% carrier ampholytes pH 6–11. PSC collagen was loaded (40 μg diluted in 8 M urea, 4% CHAPS, 1% DTE and bromophenol blue) by anodic cup loading in the Ettan™ IPGphor Cup Loading Manifold Polymeric Tray (GE Healthcare). IEF was achieved according to the following voltage steps at 16 °C: 200 V for 7 h, from 200 V to 3500 V in 2 h, 3500 V for 2 h, from 3500 V to 5000 V in 2 h, 5000 V for 3 h, from 5000 V to 8000 V in 1 h, 8000 V for 3 h, from 8000 V to 10000 V in 1 h, 10000 V until a total of 100,000 Vh was reached. The second dimension was carried out at 10 °C on 9–16% polyacrylamide linear gradient gels. Gels were stained according to a silver staining protocol compatible with MS^54^ and digitalized using an Image Scanner III (GE Healthcare). Computer-aided 2-D image analysis was carried out using ImageMaster 2-D Platinum 7.0 software (GE Healthcare).

### MALDI TOF Mass Spectrometry Analysis

Protein identification was carried out by peptide mass fingerprinting^55^,^56^ on an Ultraflex III MALDI-TOF/TOF mass spectrometer (Bruker Daltonics, Billerica, MA) as detailed in Supplementary Methods.

### Selected Reaction Monitoring (SRM) analysis

Gel pieces containing α1(I) and α2(I) were excised, washed with acetonitrile 50%, dried and re-hydrated in 10 μl of a 0.02 μg/μl sequencing-grade modified trypsin stock solution (Promega Corporation, Madison, WI, USA). Excess trypsin solution was removed and fragments submerged in 50 mM ammonium bicarbonate. Trypsin digestion proceeded overnight at 37 °C and was stopped with trifluoroacetic acid (TFA). The peptide mixture was completely dried, re-dissolved in 20 μl of 0.1% TFA in acetonitrile 2% (HPLC solvent A) and analyzed as detailed in Supplementary Methods. Out of the identified peptides that were proteotypic for α1(I) and α3(I), 4 peptides were selected for selective reaction monitoring (SRM) analysis for each protein. The 8 resulting peptides were synthesized to contain stable heavy isotopes using standard solid-phase Fmoc chemistry on a SyroI peptide synthesizer (Biotage, Uppsala, Sweden). Amino acids were coupled in a 4-fold molar excess using 1-hydroxybenzotriazole/hexafluorophosphate activation. Synthetic peptides were cleaved from the resin during 4 h using TFA (Biosolve, Valkenswaard, The Netherlands) containing 2.5% ethanedithiol and 2.5% water. Peptides were then further purified by RP-HPLC.

SRM analysis was performed on an Ultimate 3000 RSLC nano HPLC system (Thermo Fisher Scientific) coupled to a TSQ Vantage mass spectrometer (Thermo Fisher Scientific) as described in the supplementary material and methods section. Peptide amounts present in the samples were calculated using the light/heavy ratios. Each sample was analyzed in triplicate.

A high variance of this ratio between the four peptides representing the same protein was detected, which is most likely due to the variability of the (proline) hydroxylation status between the endogenous (light) peptides. This issue was tackled by using the peptide with the highest detected amount for each protein (Supplementary Table S9), assuming this peptide is either close to complete hydroxylation or close to complete non-hydroxylation and hence assuming there is less variation in the degree of hydroxylation. Ratios of α1(I) and α3(I) were calculated using these peptides. For both biological and technical replicate analysis together, the median value was calculated of the ratio α3(I)/α1(I) for each tissue type. Next the Huber scale was calculated to get an idea of the variation on the replicates. The spectral count of the unique peptides for α1(I) and α3(I) was measured (in the same samples for one biological replicate) in an LC-MS/MS run.

### Differential Scanning Calorimetry (DSC)

Collagens were further purified by several rounds of precipitation in 0.7 M NaCl, 0.5 M acetic acid, re-suspended and extensively dialyzed in 2 mM HCl or in 0.2 M Na-phosphate, 0.5 M glycerol, pH 7.4. Collagen denaturation thermograms were measured at 0.25 °C/min heating rate in a Nano III differential scanning calorimeter (Calorimetry Sciences Corporation, USA).

### Chemical characterization of collagen type I α chains

Collagen samples (27 μg) were hydrolysed in 6 M HCl at 110 °C under nitrogen and hydrolysates were derivatized with orthophthalaldelyde (OPA) and 9-fluorenyl-methyl-chloroformate (FMOC).

OPA and FMOC derivatives were analyzed by Jasco X-LC Amino Acid Analyzer with a fluorescence detector (excitation/emission at 342/456 nm for OPA-amino acids and excitation/emission at 272/312 nm for FMOC-amino acids) and by AminoQuant II amino acid analyzer (HP1090) with a diode array detector (338 nm for OPA and 262 nm for FMOC-amino acids). All results are means of three independent analyses performed on the two instruments.

### qPCR

RNA was extracted from 20 embryos for each age (0, 1, 2, 4, 4.7, 5.3, 8, 11, 13, 16, 18, 24, 48, 72, 120 hpf and 6, 8, 10, 12 dpf) using the miRNeasy mini kit (Qiagen, Germany) in combination with on-column DNase I treatment (Qiagen, Germany) according to the manufacturer’s guidelines. RNA quality index (RQI) was measured using an Experion automated electrophoresis system (software version 3.2, Bio-Rad). cDNA was synthesized from 1 μg RNA in a 20 μl reaction with the iScript kit (Bio-Rad, USA). qPCR reactions were performed and reported according to MIQE guidelines^57^. Normalization was carried out using zebrafish Expressed Repetitive Elements (ERE) (*hatn10*, *loopern4*)^58^. Expression data were analysed using qbase + software version 2.5 (Biogazelle, http://www.qbaseplus.com). We normalized the expression values to maximum expression values for each gene.

Primers for *col1a1a, col1a1b* and *col1a2* were designed using primerXL software (http://primerxl.org/). Sequences will be available upon request.

### Microarray gene expression analysis

Expression analysis was performed using custom designed 60 K Zebrafish Gene Expression Arrays (AMADID 041801; Agilent Technologies) according to the manufacturer’s instructions with 100 ng RNA as input. Normalization of these data was performed using the VSN package in R.

### *In situ* hybridization

Whole-mount *in situ* hybridization was carried out as previously described^59^. Dioxygenin uridine-5′-triphosphate (DIG) labeled RNA probes targeting *col1a1a*, *col1a1b* and *col1a2* genes were used. Further details are specified in the supplemental material and methods section. Embryos (24, 48, 72 and 96 hpf) were observed with a Leica M165 FC Fluorescent Stereo Microscope (Leica Microsystems, GmbH, Wetzlar, Germany). Four specimens for each gene were embedded in epon and cross sectioned (4 μm). Photomicrographs were acquired with a Zeiss Axioimager Z1 equipped with DIC optics and an Axiocam MRc camera^60^.

### Statistical Analysis

Statistical analysis was performed using SigmaStat 11.0. All values were expressed as mean ± standard deviation.

## Additional Information

**How to cite this article**: Gistelinck, C. *et al.* Zebrafish Collagen Type I: Molecular and Biochemical Characterization of the Major Structural Protein in Bone and Skin. *Sci. Rep.* **6**, 21540; doi: 10.1038/srep21540 (2016).

## Supplementary Material

Supplementary Information

## Figures and Tables

**Table 1:**
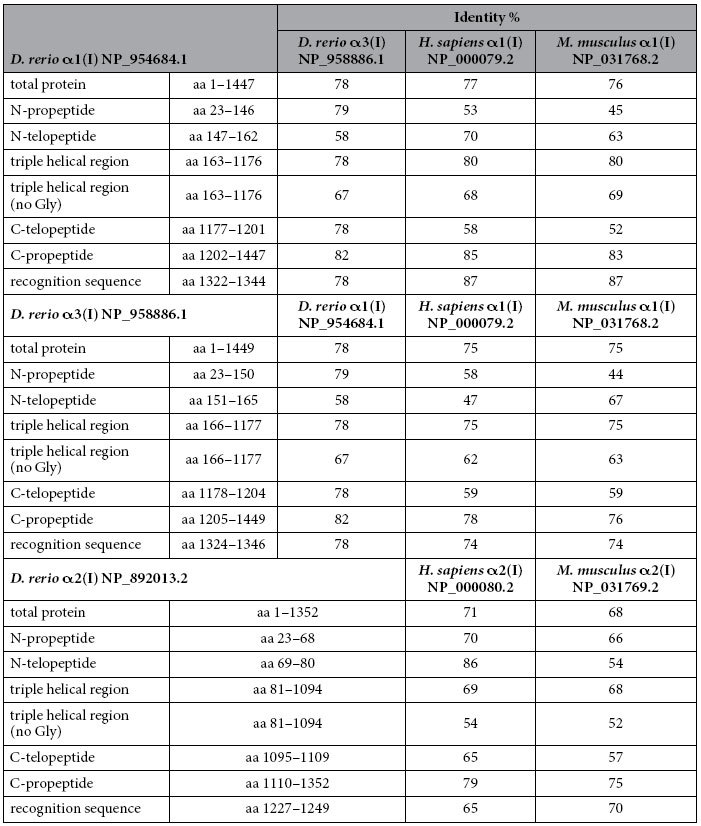
Identity analysis of type I collagen α chains between zebrafish (*D. rerio*) and human (*H. sapiens*) and mouse (*M. musculus*), respectively, following sequence alignment.

**Table 2:** Assessment of tissue specific mRNA expression for *col1a1a*, *col1a1b* and *col1a2* at 96 hpf using whole mount *in situ* hybridization followed by sectioning of the samples.

	*col1a1a*	*col1a1b*	*col1a2*
Epithelia			
epidermis	++	++	++
pectoral fin	+++	–	–
fin fold	+++	–	–
Connective tissues/fibroblasts			
connective tissue around brain	+ to ++	++	+
mesenchyme below Meckel’s cartilage	++	++	+
mandibulo-hyoideal ligament	+++	++	++
tendon of muscle sternohyoideus	+++	++	+ to ++
muscle attachment to endoskeletal disk	+	+	+
myosepta	+	++	+
Bones/osteoblasts			
parasphenoid	++	++	+
opercular	+++	++	++
cleithrum	++ to +++	++	+ to ++
ceratobranchials 1–4	+	+	+
ceratobranchial 5	++	++	+

The intensity of the detected signal is indicated as: not present (−), weak (+), average (++) or strong (+++).

**Table 3:** Theoretic and experimental Ile/Leu values.

α1:α2:α3	Ile/Leu (Theoretic Values)
2:1:0	0.770
1:1:1	0.584
0:1:2	0.398
	**Ile/Leu (Experimental Values**)
Skin	0.565 ± 0.050
Bone	0.535 ± 0.065
Scales	0.553 ± 0.097
