# Supplementary material for: Zebrafish Collagen Type I: Molecular and Biochemical Characterization of the Major Structural Protein in Bone and Skin
Source: Sci Rep. 2016 Feb 15;6:21540. doi: 10.1038/srep21540 (PMC4753508; doi:10.1038/srep21540)

Zebrafish Collagen Type I: Molecular and Biochemical Characterization of the Major Structural Protein in Bone and Skin

C. Gistelinck[1¶], R. Gioia[2¶], A. Gagliardi[3], F. Tonelli[2], L. Marchese[2], L. Bianchi[3], C. Landi[3], L. Bini[3], A. Huysseune[4], P. E. Witten[4], A. Staes[5,6], K. Gevaert[5,6], N. De Rocker[1], B. Menten[1], F. Malfait[1], S. Leikin[7], S. Carra[8], R. Tenni[2], A. Rossi[2], A. De Paepe[1], P. Coucke[1], A. Willaert[1&], A. Forlino[2*&]

[1]Center for Medical Genetics Ghent, Ghent University, Ghent, Belgium;

[2]Department of Molecular Medicine, Biochemistry Unit, University of Pavia, Pavia, Italy;

[3]Functional Proteomics Lab., Department of Life Sciences, University of Siena, Siena, Italy;

[4]Biology Department, Ghent University, Ghent, Belgium;

[5]Department of Medical Protein Research, VIB, Ghent, Belgium;

[6]Department of Biochemistry, Ghent University, Ghent, Belgium;

[7]Eunice Kennedy Shriver National Institute of Child Health and Human Development, National Institutes of Health, Bethesda, MD, USA

[8]Department of Biosciences, University of Milano, Milan, Italy;

[¶]These authors contributed equally to this work

§ These authors are shared last authors on this work.

[*] **Corresponding Author**

aforlino@unipv.it (AF)

Supplementary Methods
MALDI TOF Mass Spectrometry Analysis

Coomassie bands and silver stained spots were manually excised from the gel. Coomassie bands were destained in 2.5 mM ammonium bicarbonate and 50% (v/v) acetonitrile, while silver stained spots were destained as previously described [1]. Then, both bands and spots were dehydrated using acetonitrile. Before trypsin digestion, proteins resolved in 1D gels were reduced with 10 mM DTE in 25 mM ammonium bicarbonate, for 1 h at 56°C. Then, they were alkylated with 55 mM IAA in 25 mM ammonium bicarbonate, for 45 min at room temperature in the dark. After 10 min of incubation in 50 mM ammonium bicarbonate, protein bands were dehydrated in acetonitrile. Finally, bands and spots were rehydrated in trypsin solution (Sigma Aldrich, St. Louis, MO) and in-gel protein digestion was performed by an overnight incubation at 37°C. From each band or spot, 1.25 µl of the recovered peptide mixture was prepared for MALDI-TOF MS by spotting them onto the MALDI target, allowed to dry, and then mixed to 0.75 µl of matrix solution (saturated solution of alpha-cyano-4-hydroxycinnamic acid in 50% (v/v) acetonitrile and 0.5% (v/v) trifluoroacetic acid). Spectra were analyzed by Flex Analysis software v.3.0 and calibrated using, as internal standard, the 842.509 and 2211.105 m/z peptides arising from trypsin auto-proteolysis. The resulting mass lists were filtered for contaminant removal: mass matrix-related ions, trypsin autolysis and keratin peaks. Then, peptide mass fingerprinting searching was carried out in the NCBInr database, limited to *Danio rerio*, by Mascot (Matrix Science Ltd., London, UK, http://www.matrixscience.com). Protein identification was achieved on the basis of corresponding experimental and theoretical peptide fingerprinting patterns with a Δ mass less than 100 ppm and the number of accepted missed cleavage sites set to one. Alkylation of cysteine by carbamidomethylation was assumed as fixed modification, while oxidation of methionine and oxidation of proline were considered as possible modifications. The criteria used to accept identifications included the extent of sequence coverage, number of matched peptides, and probabilistic score.

SRM Analysis

Selected Reaction Monitoring (SRM) analysis

First, LC-MS/MS analysis was done using a LTQ-Orbitrap Velos mass spectrometer. Here, a peptide mixture was introduced into an LC-MS/MS system; the Ultimate 3000 RSLC nano (Dionex, Amsterdam, The Netherlands) in-line connected to an LTQ Orbitrap Velos (Thermo Fisher Scientific, Bremen, Germany). The sample mixture was first loaded on a trapping column (made in-house, 100 μm internal diameter (I.D.) x 20 mm, 5 μm beads C18 Reprosil-HD, Dr. Maisch, Ammerbuch-Entringen, Germany). After flushing from the trapping column, the peptides were loaded on a reverse-phase column (made in-house, 75 μm I.D. x 150 mm , 3 μm beads C18 Reprosil-HD, Dr. Maisch). Peptides were loaded with solvent A and separated with a linear gradient from 98% solvent A' (0.1% formic acid) and 2% solvent B' (0.1% formic acid and 80% acetonitrile) to 55% solvent B' at a flow rate of 300 nl/min followed by a wash reaching 100% solvent B'.

The mass spectrometer was operated in data-dependent mode, automatically switching between MS and MS/MS acquisition for the ten most abundant peaks in a given MS spectrum. In the LTQ-Orbitrap Velos, full scan MS spectra were acquired in the Orbitrap at a target value of 1E6 with a resolution of 60,000. The ten most intense ions were then isolated for fragmentation in the linear ion trap, with a dynamic exclusion of 30 s. Peptides were fragmented after filling the ion trap at a target value of 1E4 ion counts. From the MS/MS data in each LC run, Mascot Generic Files (MGFs, peak lists) were created using the Distiller software (version 2.4.3.3, Matrix Science, www.matrixscience.com/Distiller). These MGFs were then searched using the Mascot search engine (Matrix Science, www.matrixscience.com) in the Mascot Daemon interface (version 2.4, Matrix Science). Spectra were searched against the NCBI database of *zebrafish* proteins. Variable modifications were set to pyro-glutamate formation of N-terminal glutamine, acetylation of the

protein N-terminus and methionine oxidation. The mass tolerance on precursor ions was set to ± 10 ppm (with Mascot's C13 option set to 1), and on fragment ions to ± 0.5 Da. The peptide charge was set to 1+,2+,3+ and the instrument setting was put on ESI-TRAP. Enzyme was set to trypsin, allowing for one missed cleavage, and cleavage was also allowed when arginine or lysine were followed by proline. Only peptides that were ranked one and scored above the threshold score, set at 99% confidence, were withheld. All further data management was done with ms_lims [2].

The nano-LC system was configured with a trapping column (100 µm I.D. x 20 mm, 5 µm beads, C18 Reprosil-HD, Dr. Maisch) and an analytical column (75 µm I.D. x 150 mm, 3 µm beads, C18 Reprosil-HD, Dr. Maisch). Transitions for each peptide were optimized for their collision energy (CE). The 5 most intense transitions were chosen to detect the peptides. In order to approximate the concentration of the peptide in the sample, one sample was run without spike-in of the synthetic peptides. The intensities of the light peptides in the sample were used as an indication for the amount of synthetic peptide that needed to be spiked in. Spike-in concentrations can be found in Supplementary Table S9 Table. For all samples a total volume of 20 µl of synthetic peptides was added, so a final volume of 40 µl was present for analysis. From each sample, 1 µl was injected using a µl pickup method. Injection occurred at 10 µl/min in loading solvent A. After loading, the trapping column was flushed for 3 min in order to pre-concentrate the peptides while removing buffer components, before it was put in-line with the analytical column. Compounds were eluted at 300 nl/min with an acetonitrile gradient of 28 min from 4% to 55% of solvent B'. The column was washed with 90% of solvent B' for 5 min and equilibrated with solvent A' for 14 min before analysis of the next sample. The voltage on the needle in the nano-ESI source was set at 1,300 V and the capillary temperature at 275°C. A dwell time of 41 ms for each transition was applied. Transitions measured for the different peptides are listed in Supplementary Table S9. Peak areas and ratios between the heavy peptide and its light counterpart were calculated with Skyline (v2.6.1.7171, MacCoss Lab Software, University of Washington) [3].

In situ hybridization

For each WISH experiment 2 different probes were used per gene and approximately 20 embryos of the same age were stained per probe. Staining was homogenous for different embryos within one group and expression patterns for different probes detecting the same gene where highly comparable. For each gene 4 representative embryos for one of the probes were selected for embedding and sectioning. Sections of all 4 embryos per gene were assessed to determine the average signal intensity (listed in Table 2), which was comparable at the same site in each embryo hybridized for the same gene. For each gene a slide representative for the mean signal intensity at a certain site was selected for illustration in Figure 4b.

1 Gharahdaghi, F., Weinberg, C. R., Meagher, D. A., Imai, B. S. & Mische, S. M. Mass spectrometric identification of proteins from silver-stained polyacrylamide gel: a method for the removal of silver ions to enhance sensitivity. *Electrophoresis* **20**, 601-605, (1999).

2 Helsens, K. *et al.* ms_lims, a simple yet powerful open source laboratory information management system for MS-driven proteomics. *Proteomics* **10**, 1261-1264, (2010).

3 MacLean, B. *et al.* Skyline: an open source document editor for creating and analyzing targeted proteomics experiments. *Bioinformatics* **26**, 966-968, (2010).

Supplementary Table S1. List of genes flanking human *COL1A1*

Homo sapiens			***Mus musculus***			***Danio rerio***			
**Gene name**	**Ensembl ID**	**Mb from *COL1A1***	**Gene name**	**Ensembl ID**	**Mb from *Col1a1***	**Gene name**	**Ensembl ID**	**Mb from *col1a1a***	**Mb from *col1a1b***
B4GALNT2	ENSG00000167080	-1,05	*B4galnt2*	ENSMUSG00000013418	0,93	*b4galnt2.1*	ENSDARG00000094579	-0,15	
*GNGT2**	ENSG00000167083	-0,98	*Gngt2*	ENSMUSG00000038811	0,90	*gngt2a*	ENSDARG00000010680	-0,13	
						gngt2b	ENSDARG00000089997		0,17
*ABI3**	ENSG00000108798	-0,97	*Abi3*	ENSMUSG00000018381	0,89	*abi3*	ENSDARG00000060072	10,02	
						abi3	ENSDARG00000063283		2,59
PHOSPHO1	ENSG00000173868	-0,96	*Phospho1*	ENSMUSG00000050860	0,89	*phospho1*	ENSDARG00000008403	-8,10	
ZNF652	ENSG00000198740	-0,89	*Zfp652*	ENSMUSG00000075595	0,78	*znf652*	ENSDARG00000062302	-8,13	
PHB	ENSG00000167085	-0,78	*Phb*	ENSMUSG00000038845	0,73	*phb*	ENSDARG00000057414	-8,17	
*NGFR**	ENSG00000064300	-0,69	*Ngfr*	ENSMUSG00000000120	0,63	*ngfr*	ENSDARG00000088708	-3,47	
						ngfrb	ENSDARG00000032984		7,24
NXPH3	ENSG00000182575	-0,61	*Nxph3*	ENSMUSG00000046719	0,57	*nxph3*	ENSDARG00000070694	-3,22	
SPOP	ENSG00000121067	-0,58	*Spop*	ENSMUSG00000057522	0,48	*spop*	ENSDARG00000038215	-2,96	
SLC35B1	ENSG00000121073	-0,48	*Slc35b1*	ENSMUSG00000020873	0,45	*slc35b1*	ENSDARG00000038213	-2,46	
*FAM117A**	ENSG00000121104	-0,47	*Fam117a*	ENSMUSG00000038893	0,40	*fam117a*	ENSDARG00000078640	-2,44	
						fam117a	ENSDARG00000079252		7,14
*KAT7**	ENSG00000136504	-0,39	*Kat7*	ENSMUSG00000038909	0,34	*kat7*	ENSDARG00000031770		2,98
						kat7	ENSDARG00000070371	9,51	
TAC4	ENSG00000176358	-0,34	*Tac4*	ENSMUSG00000020872	0,33	/	/	/	/
*DLX4**	ENSG00000108813	-0,21	*Dlx4*	ENSMUSG00000020871	0,20	*dlx4a*	ENSDARG00000011956	11,24	
						dlx4b	ENSDARG00000071560		2,37
DLX3	ENSG00000064195	-0,19	*Dlx3*	ENSMUSG00000001510	0,18	*dlx3b*	ENSDARG00000014626		2,39
*ITGA3**	ENSG00000005884	-0,13	*Itga3*	ENSMUSG00000001507	0,11	*itga3a*	ENSDARG00000037917	11,42	
						itga3b	ENSDARG00000012824		2,44
PDK2	ENSG00000005882	-0,09	*Pdk2*	ENSMUSG00000038967	0,09	*pdk2*	ENSDARG00000020876		26,42
SAMD14	ENSG00000167100	-0,07	*Samd14*	ENSMUSG00000047181	0,07	*samd14*	ENSDARG00000090966		5,17
*PPP1R9B**	ENSG00000108819	-0,05	*Ppp1r9b*	ENSMUSG00000038976	0,05	*ppp1r9b2*	ENSDARG00000076431		6,76
						ppp1r9b	ENSDARG00000079366	0,03	
SGCA	ENSG00000108823	-0,02	*Sgca*	ENSMUSG00000001508	0,03	*sgca*	ENSDARG00000074156		-0,02
COL1A1	ENSG00000108821	0,00	***Col1a1***	ENSMUSG00000001506	0,00	***col1a1a***	ENSDARG00000012405	0,00	

							col1a1b	ENSDARG00000035809		0,00
	TMEM92	ENSG00000167105	0,09	*Tmem92*	ENSMUSG00000075610	-0,16	/	/	/	/
	XYLT2	ENSG00000015532	0,16	*Xylt2*	ENSMUSG00000020868	-0,27	*xylt2*	ENSDARG00000059557	37,07	
	MRPL27	ENSG00000108826	0,18	*Mrpl27*	ENSMUSG00000024414	-0,28	*mrpl27*	ENSDARG00000032985		19,35
	EME1	ENSG00000154920	0,19	*Eme1*	ENSMUSG00000039055	-0,29	*eme1*	ENSDARG00000076913		19,34
	LRRC59	ENSG00000108829	0,19	*Lrrc59*	ENSMUSG00000020869	-0,31	*lrrc59*	ENSDARG00000071426		29,78
	ACSF2	ENSG00000167107	0,24	*Acsf2*	ENSMUSG00000076435	-0,38	*acsf2*	ENSDARG00000061201		24,38
	CHAD	ENSG00000136457	0,28	*Chad*	ENSMUSG00000039084	-0,37	*chad*	ENSDARG00000045071		24,40
	RSAD1	ENSG00000136444	0,30	*Rsad1*	ENSMUSG00000039096	-0,40	*rsad1*	ENSDARG00000077698	24,08	
	MYCBPAP	ENSG00000136449	0,33	*Mycbpap*	ENSMUSG00000039110	-0,43	*mycbpap*	ENSDARG00000069194	1:57700573-57748929	
	EPN3	ENSG00000049283	0,35	*Epn3*	ENSMUSG00000010080	-0,45	*epn3*	ENSDARG00000078953	23,98	
	SPATA20	ENSG00000006282	0,36	*Spata20*	ENSMUSG00000020867	-0,46	*spata20*	ENSDARG00000013880		25,47
	CACNA1G	ENSG00000006283	0,38	*Cacna1g*	ENSMUSG00000020866	-0,53	*cacna1g*	ENSDARG00000089913		25,59
	ABCC3	ENSG00000108846	0,45	*Abcc3*	ENSMUSG00000020865	-0,59	*abcc3*	ENSDARG00000077243		-2,79
	ANKRD40	ENSG00000154945	0,51	*Ankrd40*	ENSMUSG00000020864	-0,61	*ankrd40*	ENSDARG00000076490	-5,72	
	LUC7L3	ENSG00000108848	0,54	*Luc7l3*	ENSMUSG00000020863	-0,65	*luc7l3*	ENSDARG00000014366	36,16	
	*WFIKKN2**	ENSG00000173714	0,65	*Wfikkn2*	ENSMUSG00000044177	-0,70	*wfikkn2a*	ENSDARG00000013460	-5,69	
							wfikkn2	ENSDARG00000059139		-2,69
	*TOB1**	ENSG00000141232	0,68	*Tob1*	ENSMUSG00000037573	-0,72	*tob1a*	ENSDARG00000032619	-5,60	
							tob1b	ENSDARG00000021372		-2,64
	*SPAG9**	ENSG00000008294	0,78	*Spag9*	ENSMUSG00000020859	-0,94	*cabz1073118.1*	ENSDARG00000078142		-2,57
							spag9a	ENSDARG00000074531	-5,48	
	NME1	ENSG00000239672	0,97	*Nme1*	ENSMUSG00000037601	-0,98	*nme2b.2*	ENSDARG00000076892	19:49813241-49861384	
	NME2	ENSG00000011052	0,97	*Gm20390*	ENSMUSG00000091228	-0,99				

*genes flanking *COL1A1* that have orthologs located in both zebrafish chromosome 3 and chromosome 12

Supplementary Table S2. List of genes flanking human *COL1A2*

	Homo sapiens			***Mus musculus***			***Danio rerio***		
	**Gene Name**	**Ensembl ID**	**Mb from *COL1A2***	**Gene name**	**Ensembl ID**	**Mb from *Col1a2***	**Gene name**	**Ensembl ID**	**Mb from *col1a2***
	AKAP9 #	ENSG00000127914	-2,45	*Akap9*	ENSMUSG00000040407	5:3928054-4080209	*cabz01010098.1*	ENSDARG00000086145	-41,47
							akap9	ENSDARG00000079610	-41,41
							cabz01010103.1	ENSDARG00000077668	-41,45
	CYP51A1 #	ENSG00000001630	-2,28	*Cyp51*	ENSMUSG00000001467	5:4081145-4104746	*cyp51*	ENSDARG00000042641	-41,37
	LRRD1	ENSG00000240720	-2,28	*Lrrd1*	ENSMUSG00000040367	5:3845173-3866596	*ascl1b*	ENSDARG00000009702	7:51153042-51174183
	KRIT1 #	ENSG00000001631	-2,20	*Krit1*	ENSMUSG00000000600	5:3803165-3844515	*krit1*	ENSDARG00000036115	5,96
	ANKIB1 #	ENSG00000001629	-2,15	*Ankib1*	ENSMUSG00000040351	5:3690000-3803109	*ankib1a*	ENSDARG00000060768	3,47
	GATAD1 #	ENSG00000157259	-1,95	*Gatad1*	ENSMUSG00000007415	5:3632932-3647934	*gatad1*	ENSDARG00000027612	3,53
	ERVW-1	ENSG00000242950	-1,93	/	/	/	/	/	/
	PEX1 #	ENSG00000127980	-1,91	*Pex1*	ENSMUSG00000005907	5:3596066-3637232	*pex1*	ENSDARG00000076647	-6,73
	RBM48 #	ENSG00000127993	-1,87	*Rbm48*	ENSMUSG00000040302	5:3583978-3596585	*rbm48*	ENSDARG00000033754	-0,81
	FAM133B #	ENSG00000234545	-1,83	*Fam133b*	ENSMUSG00000058503	5:3543844-3569810	*fam133b*	ENSDARG00000013863	-0,65
	CDK6 #	ENSG00000105810	-1,79	*Cdk6*	ENSMUSG00000040274	5:3343893-3523218	*cdk6*	ENSDARG00000070228	-0,73
	SAMD9	ENSG00000205413	-1,30	/	/	/	samd9l	ENSDARG00000095065	1:40549425-40570211
	SAMD9L	ENSG00000177409	-1,26	*Samd9l*	ENSMUSG00000047735	-1,13			
	HEPACAM2 #	ENSG00000188175	-1,21	*Hepacam2*	ENSMUSG00000044156	-1,05	*hepacam2*	ENSDARG00000061365	-0,63
	CCDC132 #	ENSG00000004766	-1,16	*Ccdc132*	ENSMUSG00000001376	-1,01	*ccdc132*	ENSDARG00000096912	-0,60
							ccdc132	ENSDARG00000002880	-0,60
	CALCR #	ENSG00000004948	-0,97	*Calcr*	ENSMUSG00000023964	-0,82	*calcr*	ENSDARG00000028845	-0,27
	GNGT1 #	ENSG00000127928	-0,80	*Gngt1*	ENSMUSG00000029663	-0,51	*gngt1*	ENSDARG00000035798	-0,13
	TFPI2 #	ENSG00000105825	-0,51	*Tfpi2*	ENSMUSG00000029664	-0,54	*tfpi2*	ENSDARG00000061351	-0,14
	GNG11 #	ENSG00000127920	-0,47	*Gng11*	ENSMUSG00000032766	-0,50	*gngt1*	ENSDARG00000035798	-0,13
	BET1 #	ENSG00000105829	-0,43	*Bet1*	ENSMUSG00000032757	-0,43	*bet1*	ENSDARG00000034885	-0,08
	COL1A2	ENSG00000164692	0,00	***Col1a2***	ENSMUSG00000029661	0,00	***col1a2***	ENSDARG00000020007	0,00
	CASD1 #	ENSG00000127995	0,11	*Casd1*	ENSMUSG00000015189	0,10	*casd1*	ENSDARG00000023900	0,02
	SGCE #	ENSG00000127990	0,19	*Sgce*	ENSMUSG00000004631	0,17	sgce	ENSDARG00000012138	0,06
	PEG10	ENSG00000242265	0,26	*Peg10*	ENSMUSG00000092035	0,24	/	/	/
	PPP1R9A #	ENSG00000158528	0,51	*Ppp1r9a*	ENSMUSG00000032827	0,40	*ppp1r9a*	ENSDARG00000061304	0,09
	PON1	ENSG00000005421	0,90	*Pon1*	ENSMUSG00000002588	0,66	*pon1*	ENSDARG00000032496	16:26885893-26895558

	PON3	ENSG00000105852	0,97	*Pon3*	ENSMUSG00000029759	0,72	*zgc:174637*	ENSDARG00000040290	16:26896251-26907913
	PON2	ENSG00000105854	1,01	*Pon2*	ENSMUSG00000032667	0,76	*pon2*	ENSDARG00000016856	16:26909176-26912212
	ASB4 #	ENSG00000005981	1,08	*Asb4*	ENSMUSG00000042607	0,88	*asb4*	ENSDARG00000034988	0,19
	PDK4 #	ENSG00000004799	1,19	*Pdk4*	ENSMUSG00000019577	0,98	*pdk4*	ENSDARG00000054848	0,21
	DYNC1I1	ENSG00000158560	1,38	*Dync1i1*	ENSMUSG00000029757	1,22	*dync1i1*	ENSDARG00000060948	16:27556254-27577115
	SLC25A13 #	ENSG00000004864	1,73	*Slc25a13*	ENSMUSG00000015112	1,54	*slc25a13*	ENSDARG00000070172	0,31
	SHFM1	ENSG00000127922	2,09	/	/	/	/	/	/
	DLX6 #	ENSG00000006377	2,61	*Dlx6*	ENSMUSG00000029754	2,36	*dlx6a*	ENSDARG00000042291	0,45
	DLX5 #	ENSG00000105880	2,63	*Dlx5*	ENSMUSG00000029755	2,37	*dlx5a*	ENSDARG00000042296	0,50
	ACN9 #	ENSG00000196636	2,72	*Acn9*	ENSMUSG00000042505	2,45	*acn9*	ENSDARG00000036092	-15,93
	TAC1 #	ENSG00000006128	3,34	*Tac1*	ENSMUSG00000061762	3,05	*tac1*	ENSDARG00000014490	-15,81
	ASNS #	ENSG00000070669	3,46	*Asns*	ENSMUSG00000029752	3,17	*asns*	ENSDARG00000016375	-15,80
	OCM2	ENSG00000135175	3,59	*Ocm*	ENSMUSG00000029618	5:144019804-144026670	*pvalb8*	ENSDARG00000037790	3:61763106-61771637
							pvalb9	ENSDARG00000071601	12:18763656-18766066
	LMTK2	ENSG00000164715	3,71	*Lmtk2*	ENSMUSG00000038970	5:144100436-144188204	*lmtk2*	ENSDARG00000076324	3:61830081-61897661
	BHLHA15	ENSG00000180535	3,82	*Bhlha15*	ENSMUSG00000052271	5:144190286-144194441	*bhlha15*	ENSDARG00000045166	12:18822354-18824381

genes flanking *COL1A2* that are located in the same chromosome of zebrafish *col1a2* (chromosome 19)

Supplementary Table S3. Content of GG+GGG and GPP repeats in human, murine and zebrafish type I collagen α chains.

	**GG + GGG**	**GPP**
H. sapiens α1(I)	3	41
H. sapiens α2(I)	3	26
M. musculus α1(I)	3	41
M. musculus α2(I)	4	21
D. rerio α1(I)	5	31
D. rerio α2(I)	6	19
D. rerio α3(I)	12	26

Supplementary Table S4. Mass spectrometry analysis of pepsin soluble collagen extracted from 48 hpf embryos and separated by 1D electrophoresis.

Band ID	Protein description	NCBI ID	Mascot search results		
			Score	No. of matched peptides	Sequence coverage (%)
A	Collagen, type I, alpha 1	gi|38649122	140	37/73	38
	Collagen alpha-1(II) chain isoform X2	gi|528487168	65	24/73	24
B	Collagen, type I, alpha 1	gi|38649122	163	25/43	24
	Collagen, type I, alpha 3	gi|42542708	117	24/43	23
C	Collagen alpha-2(I) chain precursor	gi|48762667	189	30/37	32

Supplementary Table S5. α1-α3 / α2 ratio based on densitometric analysis performed on 1D SDS-PAGE of collagen extracted from adult tissues

	**Reducing conditions***	**Non reducing conditions**
**Skin ASC**	1.89 ± 0.20	2.18 ± 0.31
**Skin PSC**	2.09± 0.44	2.00 ± 0.29
**Scales ASC**	1.79 ± 0.45	2.21 ± 0.47
**Scales PSC**	2.24 ± 0.26	2.03 ± 0.19
**Bone ASC**	2.26 ± 0.27	2.10 ± 0.21
**Bone PSC**	2.09 ± 0.43	2.16 ± 0.40

* In presence of 0.1 mM DTT

Supplementary Table S6. Mass spectrometry analysis of adult pepsin soluble collagen separated by 1D electrophoresis.

Band ID	Protein description	NCBI ID	Mascot search results		
			Score	No. of matched peptides	Sequence coverage (%)
**Bone**					
**D**	Col1a1 protein	gi|171846460	87	41/85	36
	Collagen alpha-2(I) chain precursor	gi|48762667	84	34/85	39
	Collagen, type I, alpha 3	gi|42542708	60	33/85	30
**E**	Col1a1 protein	gi|171846460	160	42/62	36
	Collagen, type I, alpha 3	gi|42542708	79	30/63	27
**F**	Collagen alpha-2(I) chain precursor	gi|48762667	150	19/20	29
**Skin**					
**D**	Col1a1 protein	gi|171846460	107	41/87	39
	Collagen alpha-2(I) chain precursor	gi|42542708	90	33/87	33
	Collagen, type I, alpha 3	gi|42542708	78	36/87	32
**E**	Col1a1 protein	gi|171846460	126	36/53	34
	Collagen, type I, alpha 3	gi|42542708	103	31/53	30
**F**	Collagen alpha-2(I) chain precursor	gi|48762667	157	19/20	29
**Scales**					
**D**	Col1a1 protein	gi|171846460	86	42/97	38
	Collagen alpha-2(I) chain precursor	gi|42542708	126	42/97	44
	Collagen, type I, alpha 3	gi|42542708	82	39/97	34
**E**	Col1a1 protein	gi|171846460	131	40/66	36
	Collagen, type I, alpha 3	gi|42542708	120	37/66	38
**F**	Collagen alpha-2(I) chain precursor	gi|48762667	144	19/21	28

Supplementary Table S7. Mass spectrometry analysis of adult pepsin soluble collagen from scales separated by 2D electrophoresis.

Spot number	Protein description	NCBI ID	Mascot search results		
			Score	No. of matched peptides	Sequence coverage (%)
**1**	Collagen alpha-2(I) chain precursor	gi|48762667	69	20/46	25
	Collagen, type I, alpha 3	gi|42542708	68	21/46	21
	Col1a1 protein	gi|171846460	67	21/46	21
**2**	Collagen, type I, alpha 3	gi|42542708	75	25/62	25
	Col1a1 protein	gi|171846460	74	24/62	28
	Collagen alpha-2(I) chain precursor	gi|48762667	66	22/62	23
**3**	Col1a1 protein	gi|171846460	102	20/31	22
	Collagen, type I, alpha 3	gi|42542708	96	19/31	19
**4**	Col1a1 protein	gi|171846460	88	18/28	23
	Collagen, type I, alpha 3	gi|42542708	88	17/28	22
**5**	Collagen, type I, alpha 3	gi|42542708	116	22/35	28
	Col1a1 protein	gi|171846460	93	22/35	26
**6**	Collagen, type I, alpha 3	gi|42542708	114	23/35	22
	Col1a1 protein	gi|171846460	102	22/35	27
**7**	Col1a1 protein	gi|171846460	161	39/64	35
	Collagen, type I, alpha 3	gi|42542708	121	34/64	33
**8**	Col1a1 protein	gi|171846460	98	22/34	22
	Collagen, type I, alpha 3	gi|42542708	94	20/34	22
**9**	Col1a1 protein	gi|171846460	111	33/53	30
	Collagen, type I, alpha 3	gi|42542708	89	27/53	27
**10**	Collagen alpha-2(I) chain precursor	gi|48762667	97	26/59	31
	Col1a1 protein	gi|171846460	74	28/59	30
	Collagen, type I, alpha 3	gi|42542708	71	26/59	27
**11**	Procollagen type I alpha 2 chain	gi|15209312	117	16/21	18
**12**	Collagen alpha-2(I) chain precursor	gi|48762667	133	34/65	34
	Collagen, type I, alpha 3	gi|42542708	89	29/65	29
**13**	Collagen alpha-2(I) chain precursor	gi|48762667	81	35/85	35
	Collagen, type I, alpha 3	gi|42542708	76	34/85	31
**14**	Collagen alpha-2(I) chain precursor	gi|48762667	152	39/72	42
	Collagen, type I, alpha 3	gi|42542708	80	30/72	29
**15**	Collagen alpha-2(I) chain precursor	gi|48762667	157	44/83	42
	Collagen, type I, alpha 3	gi|42542708	81	35/83	31
	Col1a1 protein	gi|171846460	62	32/83	31
**16**	Collagen alpha-2(I) chain precursor	gi|48762667	139	43/84	43
	Col1a1 protein	gi|171846460	77	35/84	32
	Collagen, type I, alpha 3	gi|42542708	73	33/84	30
**17**	Collagen alpha-2(I) chain precursor	gi|48762667	147	45/88	45
	Col1a1 protein	gi|171846460	74	36/88	31
**18**	Collagen alpha-2(I) chain precursor	gi|48762667	114	26/47	35
	Col1a1 protein	gi|171846460	59	21/47	24
**19**	Collagen alpha-2(I) chain precursor	gi|48762667	103	23/41	29
	Col1a1 protein	gi|171846460	63	20/41	20
**20**	Collagen alpha-2(I) chain precursor	gi|48762667	124	28/49	27
	Col1a1 protein	gi|171846460	63	20/49	22

**21**	Collagen alpha-2(I) chain precursor	gi|48762667	153	31/51	37
**22**	Collagen, type I, alpha 3<br>Col1a1 protein	gi|42542708<br>gi|171846460	159<br>158	38/67<br>41/67	38<br>40
**23**	Col1a1 protein<br>Collagen, type I, alpha 3	gi|171846460<br>gi|42542708	158<br>129	56/94<br>51/94	47<br>38
**24**	Col1a1 protein<br>Collagen, type I, alpha 3	gi|171846460<br>gi|42542708	176<br>121	59/97<br>50/97	48<br>39
**25**	Col1a1 protein<br>Collagen, type I, alpha 3	gi|171846460<br>gi|42542708	175<br>116	57/97<br>45/97	48<br>39
**26**	Col1a1 protein<br>Collagen, type I, alpha 3	gi|171846460<br>gi|42542708	181<br>86	41/57<br>27/57	37<br>27
**27**	Collagen alpha-2(I) chain precursor	gi|48762667	242	38/46	41
**28**	Collagen alpha-2(I) chain precursor	gi|48762667	285	56/73	48
**29**	Collagen alpha-2(I) chain precursor	gi|48762667	247	51/71	43
**30**	Collagen alpha-2(I) chain precursor	gi|48762667	259	33/35	34
**31**	Collagen alpha-2(I) chain precursor	gi|48762667	249	34/40	38

Supplementary Table S8. Theoretic and experimental values obtained from aminoacid analysis of zebrafish α1, α2 and α3 chains of type I collagen and type I collagen extracted from skin, bone and scales, respectively.

	Theoretic Values			
	MW	α1(I)	α2(I)	α3(I)
Asp	133,103	25	25	29
Glu	147,130	53	39	48
His	155,157	2	5	3
Cys	162,109	0	0	0
Lys	146,190	35	31	32
Arg	174,204	53	55	54
Hyp	131,131	100	84	101
Ser	105,093	31	41	43
Thr	119,120	27	21	22
Tyr	181,191	0	2	0
Hyl				
Gly	75,067	346	347	352
Asn	132,119	15	28	17
Gln	146,146	22	23	18
Ala	89,094	138	113	118
Phe	165,192	13	9	11
Ile	131,175	12	14	8
Leu	131,175	13	30	22
Met	149,208	14	12	15
Pro	115,132	103	112	108
Val	117,148	15	26	16
		1017	1017	1017

	Experimental Values		
	Skin	Bone	Scales
Asp + Asn*	45 ± 2	42 ± 1	46 ± 1
Glu + Gln*	73 ± 2	73 ± 3	76 ± 2
His	2	2 ± 1	2
Cys	0	0	0
Lys	14 ± 2	16 ±2	16 ±1
Arg	54	54	54
Hyp	112 ± 11	114 ± 22	110 ± 9
Ser	24 ± 2	21 ± 3	27 ± 2
Thr	24 ± 2	29 ± 3	30 ± 1
Tyr	2 ± 1	2	2 ± 1
Hyl	4 ± 2	5 ± 1	5 ± 1
Gly	346 ± 10	344 ± 9	338 ± 5
Ala	126 ± 2	129 ± 2	126 ± 2
Phe	13 ± 1	12 ±1	13 ±1
Ile	13 ± 1	12 ± 1	13 ± 2
Leu	23 ±1	22 ± 2	24 ± 2
Met	9 ± 1	6 ± 1	4 ± 1
Pro	113 ± 20	117 ± 16	111 ± 11
Val	20 ± 1	17 ± 1	21 ± 1
	1017	1017	1017

*due to acidic hydrolysis

Supplementary Table S9. Selective Reaction Monitoring (SRM) synthetic peptides

Protein	Synthetic Peptide Sequence	precursor m/z	fragment m/z	Collision energy (eV)	amount of heavy peptide spiked (fmol)
Type I collagen alpha 1	GAAGPPGATGFP[+16.0]GAAGR	714.355	232.14	29	8.333
Type I collagen alpha 1	GAAGPPGATGFP[+16.0]GAAGR	714.355	544.284	27	8.333
Type I collagen alpha 1	GAAGPPGATGFP[+16.0]GAAGR	714.355	748.374	29	8.333
Type I collagen alpha 1	GAAGPPGATGFP[+16.0]GAAGR	714.355	849.421	27	8.333
Type I collagen alpha 1	GAAGPPGATGFP[+16.0]GAAGR	714.355	977.48	28	8.333
Type I collagen alpha 1	GAAGPPGATGFP[+16.0]GAAGR	714.355	1.074.533	29	8.333
Type I collagen alpha 1	GAAGPPGATGFP[+16.0]GAAGR	717.362	232.14	29	8.333
Type I collagen alpha 1	GAAGPPGATGFP[+16.0]GAAGR	717.362	544.284	27	8.333
Type I collagen alpha 1	GAAGPPGATGFP[+16.0]GAAGR	717.362	748.374	29	8.333
Type I collagen alpha 1	GAAGPPGATGFP[+16.0]GAAGR	717.362	849.421	27	8.333
Type I collagen alpha 1	GAAGPPGATGFP[+16.0]GAAGR	717.362	977.48	28	8.333
Type I collagen alpha 1	GAAGPPGATGFP[+16.0]GAAGR	717.362	1.080.546	29	8.333
Type I collagen alpha 1	GFP[+16.0]GADGAAGPR	544.76	400.23	24	50
Type I collagen alpha 1	GFP[+16.0]GADGAAGPR	544.76	528.289	24	50
Type I collagen alpha 1	GFP[+16.0]GADGAAGPR	544.76	643.316	22	50
Type I collagen alpha 1	GFP[+16.0]GADGAAGPR	544.76	714.353	23	50
Type I collagen alpha 1	GFP[+16.0]GADGAAGPR	544.76	884.422	16	50
Type I collagen alpha 1	GFP[+16.0]GADGAAGPR	547.767	406.244	24	50
Type I collagen alpha 1	GFP[+16.0]GADGAAGPR	547.767	534.303	24	50
Type I collagen alpha 1	GFP[+16.0]GADGAAGPR	547.767	649.33	22	50
Type I collagen alpha 1	GFP[+16.0]GADGAAGPR	547.767	720.367	23	50
Type I collagen alpha 1	GFP[+16.0]GADGAAGPR	547.767	890.436	16	50
Type I collagen alpha 1	GNPGAEGATGPAGIP[+16.0]GPQGIGGQR	1.066.528	417.22	40	100
Type I collagen alpha 1	GNPGAEGATGPAGIP[+16.0]GPQGIGGQR	1.066.528	982.506	40	100
Type I collagen alpha 1	GNPGAEGATGPAGIP[+16.0]GPQGIGGQR	1.066.528	1.152.612	40	100
Type I collagen alpha 1	GNPGAEGATGPAGIP[+16.0]GPQGIGGQR	1.066.528	1.320.702	40	100

Type I collagen alpha 1	GNPGAEGATGPAGIP[+16.0]GPQGIGGQR	1.066.528	1.377.723	40	100
Type I collagen alpha 1	GNPGAEGATGPAGIP[+16.0]GPQGIGGQR	1.071.532	427.229	40	100
Type I collagen alpha 1	GNPGAEGATGPAGIP[+16.0]GPQGIGGQR	1.071.532	992.515	40	100
Type I collagen alpha 1	GNPGAEGATGPAGIP[+16.0]GPQGIGGQR	1.071.532	1162.62	40	100
Type I collagen alpha 1	GNPGAEGATGPAGIP[+16.0]GPQGIGGQR	1.071.532	1330.71	40	100
Type I collagen alpha 1	GNPGAEGATGPAGIP[+16.0]GPQGIGGQR	1.071.532	1.387.732	40	100
Type I collagen alpha 1	VGPPGP[+16.0]SGNSGP[+16.0]P[+16.0]GPPGPAGK	914.945	147.113	35	41.666
Type I collagen alpha 1	VGPPGP[+16.0]SGNSGP[+16.0]P[+16.0]GPPGPAGK	914.945	204.134	35	41.666
Type I collagen alpha 1	VGPPGP[+16.0]SGNSGP[+16.0]P[+16.0]GPPGPAGK	914.945	372.224	35	41.666
Type I collagen alpha 1	VGPPGP[+16.0]SGNSGP[+16.0]P[+16.0]GPPGPAGK	914.945	623.351	34	41.666
Type I collagen alpha 1	VGPPGP[+16.0]SGNSGP[+16.0]P[+16.0]GPPGPAGK	914.945	906.468	35	41.666
Type I collagen alpha 1	VGPPGP[+16.0]SGNSGP[+16.0]P[+16.0]GPPGPAGK	918.952	155.127	35	41.666
Type I collagen alpha 1	VGPPGP[+16.0]SGNSGP[+16.0]P[+16.0]GPPGPAGK	918.952	212.148	35	41.666
Type I collagen alpha 1	VGPPGP[+16.0]SGNSGP[+16.0]P[+16.0]GPPGPAGK	918.952	380.238	35	41.666
Type I collagen alpha 1	VGPPGP[+16.0]SGNSGP[+16.0]P[+16.0]GPPGPAGK	918.952	631.365	34	41.666
Type I collagen alpha 1	VGPPGP[+16.0]SGNSGP[+16.0]P[+16.0]GPPGPAGK	918.952	914.482	35	41.666
Type I collagen alpha 3	GFGGLAGPSGEPGK	615.809	204.134	26	0.5
Type I collagen alpha 3	GFGGLAGPSGEPGK	615.809	301.187	26	0.5
Type I collagen alpha 3	GFGGLAGPSGEPGK	615.809	671.336	19	0.5
Type I collagen alpha 3	GFGGLAGPSGEPGK	615.809	728.357	19	0.5
Type I collagen alpha 3	GFGGLAGPSGEPGK	615.809	799.394	19	0.5
Type I collagen alpha 3	GFGGLAGPSGEPGK	619.318	204.134	26	0.5
Type I collagen alpha 3	GFGGLAGPSGEPGK	619.318	301.187	26	0.5
Type I collagen alpha 3	GFGGLAGPSGEPGK	619.318	671.336	19	0.5
Type I collagen alpha 3	GFGGLAGPSGEPGK	619.318	728.357	19	0.5
Type I collagen alpha 3	GFGGLAGPSGEPGK	619.318	799.394	19	0.5
Type I collagen alpha 3	GGAGPP[+16.0]GATGFP[+16.0]GAAGR	715.345	232.14	28	37.6
Type I collagen alpha 3	GGAGPP[+16.0]GATGFP[+16.0]GAAGR	715.345	544.284	28	37.6
Type I collagen alpha 3	GGAGPP[+16.0]GATGFP[+16.0]GAAGR	715.345	748.374	27	37.6
Type I collagen alpha 3	GGAGPP[+16.0]GATGFP[+16.0]GAAGR	715.345	849.421	26	37.6
Type I collagen alpha 3	GGAGPP[+16.0]GATGFP[+16.0]GAAGR	715.345	1.090.528	29	37.6
Type I collagen alpha 3	GGAGPP[+16.0]GATGFP[+16.0]GAAGR	718.351	232.14	28	37.6

Type I collagen alpha 3	GGAGPP[+16.0]GATGFP[+16.0]GAAGR	718.351	544.284	28	37.6
Type I collagen alpha 3	GGAGPP[+16.0]GATGFP[+16.0]GAAGR	718.351	748.374	27	37.6
Type I collagen alpha 3	GGAGPP[+16.0]GATGFP[+16.0]GAAGR	718.351	849.421	26	37.6
Type I collagen alpha 3	GGAGPP[+16.0]GATGFP[+16.0]GAAGR	718.351	1.090.528	29	37.6
Type I collagen alpha 3	GNDGNTGAAGP[+16.0]PGPTGPAGPP[+16.0]GFP[+16.0]GGAGAK	1.275.086	147.113	45	1000
Type I collagen alpha 3	GNDGNTGAAGP[+16.0]PGPTGPAGPP[+16.0]GFP[+16.0]GGAGAK	1.275.086	275.171	44	1000
Type I collagen alpha 3	GNDGNTGAAGP[+16.0]PGPTGPAGPP[+16.0]GFP[+16.0]GGAGAK	1.275.086	346.208	42	1000
Type I collagen alpha 3	GNDGNTGAAGP[+16.0]PGPTGPAGPP[+16.0]GFP[+16.0]GGAGAK	1.275.086	573.299	45	1000
Type I collagen alpha 3	GNDGNTGAAGP[+16.0]PGPTGPAGPP[+16.0]GFP[+16.0]GGAGAK	1.275.086	987.489	46	1000
Type I collagen alpha 3	GNDGNTGAAGP[+16.0]PGPTGPAGPP[+16.0]GFP[+16.0]GGAGAK	1.279.093	155.127	45	1000
Type I collagen alpha 3	GNDGNTGAAGP[+16.0]PGPTGPAGPP[+16.0]GFP[+16.0]GGAGAK	1.279.093	283.186	44	1000
Type I collagen alpha 3	GNDGNTGAAGP[+16.0]PGPTGPAGPP[+16.0]GFP[+16.0]GGAGAK	1.279.093	354.223	42	1000
Type I collagen alpha 3	GNDGNTGAAGP[+16.0]PGPTGPAGPP[+16.0]GFP[+16.0]GGAGAK	1.279.093	581.313	45	1000
Type I collagen alpha 3	GNDGNTGAAGP[+16.0]PGPTGPAGPP[+16.0]GFP[+16.0]GGAGAK	1.279.093	995.504	46	1000
Type I collagen alpha 3	VGP[+16.0]PGPSGASGPPGPTGP[+16.0]AGK	887.442	204.134	35	3.57
Type I collagen alpha 3	VGP[+16.0]PGPSGASGPPGPTGP[+16.0]AGK	887.442	388.219	35	3.57
Type I collagen alpha 3	VGP[+16.0]PGPSGASGPPGPTGP[+16.0]AGK	887.442	445.241	35	3.57
Type I collagen alpha 3	VGP[+16.0]PGPSGASGPPGPTGP[+16.0]AGK	887.442	894.468	35	3.57
Type I collagen alpha 3	VGP[+16.0]PGPSGASGPPGPTGP[+16.0]AGK	887.442	1.038.521	35	3.57
Type I collagen alpha 3	VGP[+16.0]PGPSGASGPPGPTGP[+16.0]AGK	891.449	212.148	35	3.57
Type I collagen alpha 3	VGP[+16.0]PGPSGASGPPGPTGP[+16.0]AGK	891.449	396.233	35	3.57
Type I collagen alpha 3	VGP[+16.0]PGPSGASGPPGPTGP[+16.0]AGK	891.449	453.255	35	3.57
Type I collagen alpha 3	VGP[+16.0]PGPSGASGPPGPTGP[+16.0]AGK	891.449	902.482	35	3.57
Type I collagen alpha 3	VGP[+16.0]PGPSGASGPPGPTGP[+16.0]AGK	891.449	1.046.536	35	3.57

Peptides selected for spike-in are highlighted in yellow.

[+16.0] in the peptide sequence indicates the hydroxylation of the Proline residue

Supplementary Figure 1. Expression of collagen type I genes in early zebrafish development. Relative expression values were normalized to maximum expression values for each gene and depicted in the y-axis. Only low levels of maternal transcripts can be detected for the three collagen type I genes. An expression peak for all three genes is detected at 72-96 hpf.

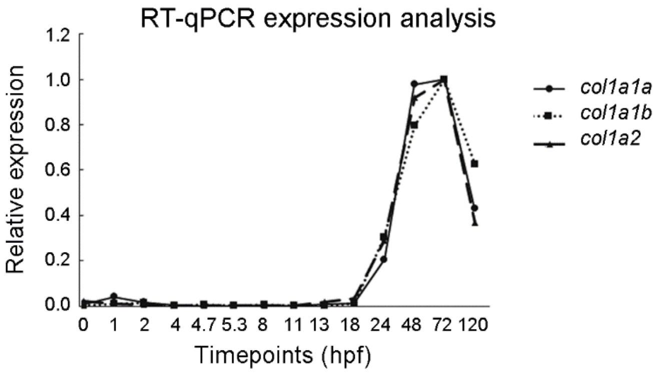

Supplement: Supplementary Information [file srep21540-s1.pdf]
